# Caveolin-1 Protects B6129 Mice against *Helicobacter pylori* Gastritis

**DOI:** 10.1371/journal.ppat.1003251

**Published:** 2013-04-11

**Authors:** Ivana Hitkova, Gang Yuan, Florian Anderl, Markus Gerhard, Thomas Kirchner, Simone Reu, Christoph Röcken, Claus Schäfer, Roland M. Schmid, Roger Vogelmann, Matthias P. A. Ebert, Elke Burgermeister

**Affiliations:** 1 Department of Medicine II, Universitätsmedizin Mannheim, Medical Faculty Mannheim, Heidelberg University, Mannheim, Germany; 2 Department of Gastroenterology, The First Affiliated Hospital of Sun Yat-sen University, Guangzhou, People's Republic of China; 3 Institute of Medical Microbiology, Immunology and Hygiene, Technische Universität München, München, Germany; 4 German Centre for Infection Research (DZIF), München, Germany; 5 Institute of Pathology, Klinikum der Universität München, München, Germany; 6 Department of Pathology, Christian-Albrechts Universität, Kiel, Germany; 7 Department of Medicine II, Klinikum der Universität München, München, Germany; 8 Department of Medicine II, Klinikum rechts der Isar, Technische Universität München, München, Germany; University of Illinois, United States of America

## Abstract

Caveolin-1 (Cav1) is a scaffold protein and pathogen receptor in the mucosa of the gastrointestinal tract. Chronic infection of gastric epithelial cells by *Helicobacter pylori* (*H. pylori*) is a major risk factor for human gastric cancer (GC) where Cav1 is frequently down-regulated. However, the function of Cav1 in *H. pylori* infection and pathogenesis of GC remained unknown. We show here that Cav1-deficient mice, infected for 11 months with the CagA-delivery deficient *H. pylori* strain SS1, developed more severe gastritis and tissue damage, including loss of parietal cells and foveolar hyperplasia, and displayed lower colonisation of the gastric mucosa than wild-type B6129 littermates. Cav1-null mice showed enhanced infiltration of macrophages and B-cells and secretion of chemokines (RANTES) but had reduced levels of CD25^+^ regulatory T-cells. Cav1-deficient human GC cells (AGS), infected with the CagA-delivery proficient *H. pylori* strain G27, were more sensitive to CagA-related cytoskeletal stress morphologies (“humming bird”) compared to AGS cells stably transfected with Cav1 (AGS/Cav1). Infection of AGS/Cav1 cells triggered the recruitment of p120 RhoGTPase-activating protein/deleted in liver cancer-1 (p120RhoGAP/DLC1) to Cav1 and counteracted CagA-induced cytoskeletal rearrangements. In human GC cell lines (MKN45, N87) and mouse stomach tissue, *H. pylori* down-regulated endogenous expression of Cav1 independently of CagA. Mechanistically, *H. pylori* activated sterol-responsive element-binding protein-1 (SREBP1) to repress transcription of the human Cav1 gene from sterol-responsive elements (SREs) in the proximal Cav1 promoter. These data suggested a protective role of Cav1 against *H. pylori*-induced inflammation and tissue damage. We propose that *H. pylori* exploits down-regulation of Cav1 to subvert the host's immune response and to promote signalling of its virulence factors in host cells.

## Author Summary

Infection with the bacterium *Helicobacter pylori* (*H. pylori*) mainly affects children in the developing countries who are at risk to progress to gastric cancer (GC) as adults after many years of persistent infection, especially with strains which are positive for the oncogenic virulence factor CagA. Eradication of *H. pylori* by antibiotics is a treatment of choice but may also alter the susceptibility to allergies and other tumor types. Thus, novel diagnostic or prognostic markers are needed which detect early molecular changes in the stomach mucosa during the transition of chronic inflammation to cancer. In our study, we found that the tumor suppressor caveolin-1 (Cav1) is reduced upon infection with *H. pylori*, and CagA was sufficient but not necessary for this down-regulation. Loss of Cav1 was caused by *H. pylori*-dependent activation of sterol-responsive element-binding protein-1 (SREBP1), and this event abolished the interaction of Cav1 with p120 RhoGTPase-activating protein/deleted in liver cancer-1 (p120RhoGAP/DLC1), a second *bona fide* tumor suppressor in gastric tissue. Conclusively, Cav1 and DLC1 may constitute novel molecular markers in the *H. pylori*-infected gastric mucosa before neoplastic transformation of the epithelium.

## Introduction

*Helicobacter pylori* (*H. pylori*) is a Gram-negative bacterium which colonizes stomachs of approx. 50% of the world's population and increases the risk for development of chronic gastritis, peptic ulcer disease, gastric mucosa-associated lymphoid tissue (MALT) lymphoma, mucosal atrophy and gastric cancer (GC) [1], [2]. Based on this etiology, *H. pylori* has been classified as a class I carcinogen by the World Health Organisation (WHO) in 1994 [3].

The two major *H. pylori* toxins [4], CagA and VacA, are internalized into gastric epithelial cells by injection via the bacterial type IV secretion system (CagA) [5] or by direct insertion into lipid rafts (VacA) [6], [7]. Lipid rafts are cholesterol and sphingolipid-rich microdomains of the plasma membrane [8], [9] which are exploited by many pathogens, including viruses, parasites and bacteria, to facilitate uptake of whole organisms and/or internalisation of toxins into host cells [10], [11], [12]. For example, *Neisseria spec*. uses lipid rafts and Rho-mediated signaling of the actin cytoskeleton to gain access to the cytosol [13]. *Pseudomonas aeruginosa* exploits lipid raft-associated toll-like receptor 2 for infection of lung epithelial cells [14].

Caveolin-1 (Cav1) is the 21–24 kDa major and essential structural protein of caveolae, a specialized form of lipid raft microdomains. Caveolae are 50–100 nm flask/tube-shaped invaginations of the plasma membrane abundant in macrophages, endothelial and smooth muscle cells, type I pneumocytes and adipocytes, where they participate in cellular transport processes including endocytosis, cholesterol efflux and membrane traffic [15], [16]. In this context, Cav1 can also act as an inhibitor of clathrin-independent endocytosis and block pathogen/toxin uptake [17], [18]. Through binding to its scaffolding domain, Cav1 directly inhibits a plethora of receptors and enzymes including tyrosine kinases of the Src and Ras family, G-proteins and nitric oxide synthases [15]. In addition to a role in membrane traffic, Cav1 thus constitutes a control platform for regulation of cell proliferation and survival [19]. Cav1 also exerts an important function in cell motility and migration and, within epithelial, stromal and endothelial tissues, by enforcing cell-cell contacts, cell-matrix adhesion and immune responses [20], [21], [22], [23].

Cav1 directly binds cholesterol, and transcription of Cav1 is negatively regulated by the transcription factor sterol-responsive element-binding protein-1 (SREBP1) [24]. SREBP1 is bound to the endoplasmic reticulum (ER) as an inactive 125 kDa precursor and is activated under conditions of cholesterol deficiency by proteolytic cleavage in the Golgi apparatus. This cleavage is followed by translocation of the active 68 kDa SREBP1 into the nucleus where it binds to sterol-responsive elements (SREs) of target genes, including Cav1, involved in synthesis of cholesterol and fatty acids [25]. *H. pylori* has been shown to metabolize cholesterol from the host cell membrane, and host cholesterol alters the oncogenic properties of CagA [26], [27].

We therefore hypothesized that the cholesterol-binding proteins SREBP1 and Cav1 are targets of *H. pylori* infection and/or effector functions. Specifically, we asked whether (i) *H. pylori* exploits Cav1 to facilitate injection and down-stream signalling of CagA in gastric epithelial cells or (ii) Cav1 acts as a protective “barrier-enforcing” protein that counteracts disease evoked by *H. pylori*. To test this, the phenotypes which result from *H. pylori* infection were studied in Cav1-deficient mice and in human GC cell lines. Our data showed that Cav1 protected B6129 mice against *H. pylori*-related gastritis and tissue damage *in vivo* independently of CagA. *H. pylori* also activated SREBP1 and down-regulated expression of murine and human Cav1 independently of CagA. In addition, Cav1 counteracted CagA-dependent cytoskeletal rearrangements *in vitro* by recruitment of the tumor suppressor deleted in liver cancer-1 (DLC1).

## Materials and Methods

### Ethics statement

Animal studies were conducted in agreement with the ethical guidelines of the Technische Universität München (German Animal Welfare Act, Deutsches Tierschutzgesetz) and had been approved (#55.2-1-54-2531-74-08) by the government of Bavaria (Regierung von Obb., Munich, Germany).

### Animals

Homozygous Cav1 knockout (Cav1-KO) (strain Cav1tm1Mls/J; stock number 004585) and matched control wild-type (WT) (strain B6129SF2/J; stock number 101045) mice (8 weeks) were obtained from the Jackson Laboratory (Bar Harbor, Maine) and maintained on a mixed background in a pathogen-free mouse facility [28], [29]. Experimental gastric ulceration was performed with indomethacin as published before [30]. Infection of mice with the mouse-adapted CagA/VacA-delivery deficient *H. pylori* strain SS1 was performed by oral gavage as described [31]. The average time mice from different genetic backgrounds (C57BL/6, B6129, BALB/c) take to progress to chronic gastritis and beyond (gastric atrophy, hyperplasia, dysplasia) [32] ranges between 10 and 15 month upon infection with the standardized reference strain SS1 [28], [33], [34], [35]. We therefore decided to perform our analysis within this time frame.

### Reagents

Chemicals were from Merck (Darmstadt, Germany) or Sigma (Taufkirchen, Germany). Polyclonal antisera were SREBP1 (#PA1-46142, Thermofisher Scientific, Waltham, MA), Cav1 (N-20, sc-894), SREBP1 (C-20, sc-366), CagA (b-300, sc-25766), FAK (A-17, sc-557), phospho-FAK (Tyr-397, sc-11765), Hsp90 alpha/beta (H-114, sc-7947), Lamin A/C (H-110, sc-20681, all from Santa Cruz Biotech., CA), general and phospho ERK1/2 (p44/p42), p38, JNK (all from Cell Signaling, Danvers, MA) and Ki-67 (SP6, DCS GmbH, Hamburg, Germany). Mouse monoclonal antibodies were Cav1 (#610406) and phospho-Cav1 (pY14, #611338) (both from BD/Transduction Lab., San Jose, CA), DLC-1 (C-12, sc-271915) and beta-Actin (AC-15, sc-69879) (both from Santa Cruz Biotech.). The macrophage-specific rat anti-mouse F4/80 antibody (#MF48000) was obtained from Invitrogen (Life Technologies, Darmstadt, Germany). Chicken anti-*H. pylori* polyclonal Ab was used as described [36]. Serum cytokines were measured by ELISA (R&D Systems, Minneapolis, MN) according to the manufacturer's instructions. Pull-down assays for the small GTPases Rho/Rac/Cdc42 were purchased from Biocat (Heidelberg, Germany).

### Cell culture

Human embryonic kidney (HEK293), Madin-Darby canine kidney (MDCK), parental human GC cell lines (AGS, MKN45, N87) (all from the American Type Culture Collection, Rockville, MD) and stably transfected clones generated thereof were maintained as described previously [37]. Infection of cells with the cell-adapted CagA-delivery proficient *H. pylori* strain G27 was performed as before [36].

### DNA-constructs

The expression plasmid pEGFP-CagA was mentioned elsewhere [38]. The ∼800 bp fragment of the proximal human Cav1 promoter (AF019742, position 69 to 859) [24] was amplified by PCR from the genomic DNA of human normal liver and cloned into the KpnI/HindIII sites of pGL3-luc luciferase reporter plasmid (Promega GmbH, Mannheim, Germany). Isoform 4 of the human DLC1 mRNA [39] (DLC1v4, NM_001164271.1) was amplified from human hepatoma HepG2 cells and inserted in the BamHI/NotI sites of the expression vector pTarget (pT, Promega GmbH). Transient transfection and luciferase assays were performed as before [37].

### Bacterial culture

*H. pylori* SS1 and G27 bacteria were recovered from −80°C glycerol stocks and grown on Wilkins-Chalgren (WC) blood agar plates under microaerobic conditions (10% CO2, 5% O2, 85% N2; 37°C) for 2–3 days. The mouse-adapted *H. pylori* SS1 was harvested from agar plates for *in vivo* infections as published previously [31]. The SS1 strain was PCR-positive for the *cagA* gene and mRNA but did not inject functional CagA protein [40] as evident by the absence of the “humming bird” phenotype in infected AGS cells (data not shown). The cell-adapted *H. pylori* bacteria CagA-delivery proficient G27 *wt* and the CagA-deletion mutant G27 *Delta cagA* were harvested from agar plates and subsequently grown in continuous co-culture with MDCK cells as described [36].

### *Ex vivo* quantification of colony forming units (CFUs)

Whole stomachs were excised from mice, and colony formation was determined essentially as described [31]. An antral strip of the stomach was weighed, placed into 5 ml of Brucella broth and vortexed for 10 min. Dilutions of 1∶10, 1∶100 and 1∶1000 were prepared, and 100 µl of each dilution was plated onto *H. pylori*-selective WC blood agar plates. The number of bacterial colonies was determined after 5 days and normalised to the weight of the corresponding stomach pieces.

### Processing of mouse gastric tissue

The remaining stomach was washed with sterile water. An antral strip was cut, frozen in liquid nitrogen and stored at −80°C until RNA extraction. The rest of the stomach was placed into 3 ml of 4% (w/v) paraformaldehyde (PFA) in phosphate buffered saline (PBS) and incubated for 24 h at 4°C. Then, the stomach was cut along the greater and small curvature into two halves, followed by dehydration and embedding into paraffin for histological analysis.

### Gentamycin protection assay

Cells were infected with the *H. pylori* G27 strain for 2 to 24 h at a multiplicity of infection (MOI) of 500∶1. Thereafter, cells were washed three times with PBS to remove residual bacteria and were additionally incubated for 2 h at 37°C in a humidified atmosphere in DMEM/F12 (10% FCS, 10% Brucella broth) supplemented with gentamycin (200 µg/ml), penicillin/streptomycin (100 µg/ml) and chloramphenicol (100 µg/ml). Absence of extracellular bacteria was confirmed under the microscope, and the cells were subsequently lysed for detection of intracellular CagA by Western blot (WB).

### Coimmunoprecipitation (CoIP) and Western blot (WB)

Detection of immunoprecipitated proteins by SDS-PAGE and WB was performed as before [41]. Matrix-assisted Laser Desorption/Ionization mass spectrometry (MALDI-MS) was described in detail in [29].

### Immunofluorescence

The staining was performed in triple-color mode visualizing 4,6-diamidino-2-phenylindole (DAPI), Alexa-488 and -594 using a digital camera-connected (Axiovision, release 4.4) fluorescence microscope (Axiovert 200M, Carl Zeiss MicroImaging GmbH, Hallbergmoos, Germany). Confocal microscopy (Axiovert 40, Zeiss) and 3D-reconstruction of *H. pylori*-infected cells with LSM510 (Zeiss) and Volocity (Improvision, Tübingen, Germany) was done as before [37].

### Histopathological evaluation and immunohistochemistry (IHC)

Chronic active gastritis was defined by the simultaneous presence of both neutrophilic polymorphnuclear (PMN) and mononuclear cells (lymphocytes and plasma cells) within the gastric mucosa. Active (PMN) and chronic (mononuclear) infiltrate was assessed as follows: Paraffin-embedded gastric tissue was cut into 3 µm sections using a semi-automatic microtome (Leica Microsystems GmbH, Wetzlar, Germany). The sections were then stained using Hematoxylin & Eosin (H&E) solutions. The histopathological analysis was carried out by three pathologists (CR, SR, TK) blinded to the study setup. Morphological alterations in the gastric mucosa were classified according to the updated Sydney system [32], [42]. The grade of gastritis was scored based on the density of intramucosal inflammatory infiltrates from mononuclear and PMN cells as published before [43]: none (0), mild (1+), moderate (2+) and severe (3+). In addition, hyperplastic or regenerative epithelial alterations, loss of parietal cells and the frequency of lymphoid follicles or lymphoid aggregates were noted. The intensity of *H. pylori* colonization in the gastric mucosa was recorded as mild (few and single bacteria in a random distribution), moderate (single and clustered bacteria in a discontinuous distribution) and severe (dense bacterial clusters covering the gastric mucosa in continuous layers). Multiple scores of different regions of the stomach were determined. Immunohistochemistry (IHC) was performed on paraffin sections as described before [44].

### Electrophoretic mobility shift assay (EMSA), chromatin immunoprecipitation (ChIP), reverse transcription PCR (RT-PCR) and quantitative PCR (qPCR)

ChIP (Kit from Upstate, Millipore GmbH, Schwalbach, Germany) and all other methods were performed as described previously [45]. Oligonucleotides are listed in **Table S1**.

### Cellular assays

Viability of adherent cells was measured by 1-(4,5-dimethylthiazol-2-yl)3,5-diphenyl-formazan (MTT) assay (Roche Diagnostics GmbH, Mannheim, Germany) as recommended by the manufacturer. To determine cell adhesion, 1×10^4^ cells were seeded into 6 cm cell culture dishes for 1 to 6 h followed by repetitive washing with PBS. The remaining adherent cells were fixed with 4% (w/v) PFA in PBS, stained with crystal violet and subsequently counted using ImageJ (NIH, Bethesda, MD). Wound healing assays were performed essentially as described in [46]. Briefly, cells were grown to confluence in 6 cm dishes, and a 5 mm scratch was introduced into the monolayer using an inverted blue tip followed by incubation of the cell culture plates for additional 24, 48 and 72 h. Wound closure was monitored upon fixation and staining of cells with crystal violet using bright field microscopy (Axiovert 200M, Carl Zeiss MicroImaging GmbH).

### Statistics

Results are means ± S.E. from at least 5 animals per genotype or 3 independent experiments from different cell passages. The software GraphPad Prism (version 4.0, La Jolla, CA) was used to analyze the data. P-values (*p<0.05) were calculated using Student's t and Fisher Exact tests.

### Accession numbers

Human: Cav1: NM_001753.4, Q03135; b2M: NM_004048.2, P61769; IL8: NM_000584.3, P10145; DLC1 v1: NM_182643.2, Q96QB1; DLC1 v4: NM_001164271.1, Q96QB1; ACS: NM_018677.3, Q9NR19; HMGCoAS: NM_001098272.2, Q01581; HMGCoAR: NM_000859.2, P04035; LDLR: NM_000527.4, P01130; beta-Actin: P60709; Lamin A: P02545; Lamin C: P02545; Hsp90 alpha: P07900; Hsp90 beta: P08238; ERK1 (p44): P27361; ERK2 (p42): P28482; FAK: Q05397; JNK1: P45983; JNK2: P45984; p38: Q16539; Src: P12931; SREBP1: P36956; Ki-67: P46013; Mouse: Cav1: NM_007616.4, P49817; b2M: NM_009735.3, Q91XJ8; TNFalpha: NM_013693.2, P06804; IFNgamma: NM_008337.3, P01580; IL1beta: NM_008361.3, P10749; IL6: NM_031168.1, P08505; CD4: NM_013488.2, P06332; CD19: NM_009844.2, P25918; CD25: NM_000417.2, P01589; CD86: NM_019388.3, P42082; CCL5: NM_013653.3, P30882; CXCL1: NM_008176.3, P12850; PPARg2: NM_015869.4, P37231; TFF2: NM_009363.3, Q9QX97; Dog: b2M: NC_006612, XP_850148; *H. pylori*: CagA: YP_002266135.1, B5Z6S0; UreB: YP_626814.1, Q1CV82.

## Results

### Cav1-deficient mice display enhanced gastritis upon infection with CagA-delivery incompetent *H. pylori* SS1

**Figure 1:**
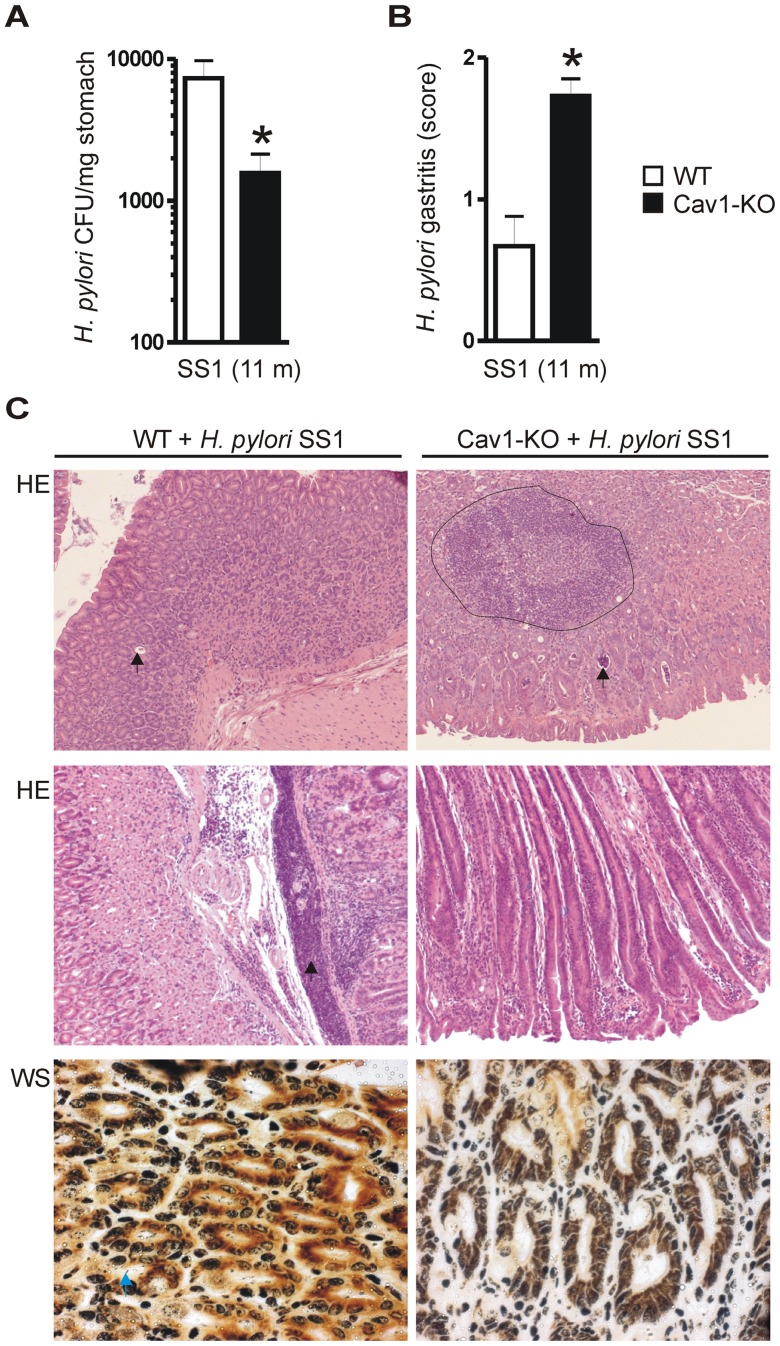
Cav1-deficient mice show enhanced gastritis upon infection with *H. pylori* SS1. B6129 WT and Cav1-KO mice (n = 15 per group) were infected with the mouse-adapted CagA-delivery incompetent *H. pylori* SS1 strain for 11 month. (A) Stomachs of Cav1-KO mice were less colonized by SS1 bacteria than those of WT mice. *Ex vivo* quantification of colony forming units (CFUs) per mg of resected stomach tissue. The data are presented as mean ± S.E. (n = 15 per group); *p = 0.0141 KO *versus* WT. (B–C) Hematoxylin & Eosin (H&E) and Warthin-Starry silver (WS) stainings from paraffin sections of mouse gastric tissue were evaluated for signs of histopathology and results were scored for individual mice (0+ no, 1+ mild , 2+ moderate, 3+ severe gastritis). Data are presented as in (A) (n = 15 per group); *p = 0.0002 KO *versus* WT. Cav1-KO mice show active chronic gastritis with intramucosal lymphocyte follicles, foveolar hyperplasia and loss of parietal cells. Quantitative analyses (B) are shown together with representative histology (C); magnifications 100×, 200×.

To assess the histological changes induced in gastric tissue upon *H. pylori* infection, B6129 WT and Cav1-KO mice were infected with the mouse-adapted and CagA-delivery deficient *H. pylori* strain SS1. The mice were euthanized 11 months later, and *H. pylori* was isolated from resected stomach tissue [31]. Cav1-KO mice showed less bacterial colonisation of the gastric mucosa than WT mice (7.3±2.4 WT *versus* 1.6±0.5 KO ×10^3^ CFU/mg stomach tissue; *p = 0.0141; n = 15 per genotype) (**Fig. 1A**). Histopathological analysis revealed that both WT and Cav1-KO mice developed active chronic gastritis accompanied by infiltration of mononuclear and polymorphnuclear (PMN) cells into the gastric mucosa (**Fig. 1B**). In contrast, uninfected WT and Cav1-KO mice had no intramucosal inflammation (data not shown). Instead, the gastritis was markedly enhanced in *H. pylori*-infected Cav1-KO mice compared with infected WT mice (**Fig. 1C**). In Cav1-KO mice, the average score of gastritis (0.7±0.2 WT *versus* 1.7±0.1 KO; *p = 0.0002, n = 15 per genotype) was more severe (**Table 1**) than in WT mice, and the stomach mucosa exhibited intramucosal B-cell follicles, foveolar hyperplasia and loss of parietal cells. This data indicate that Cav1-deficiency is associated with an increased inflammatory response in the gastric mucosa and a less efficient colonisation by *H. pylori*.

**Table 1:** Histopathology of *H. pylori* gastritis.

	*Cav1 **−/−***	*Cav1 **+/+***
Score 0	0/15	8/15
Score 1+	4/15	4/15
Score 2+	11/15	3/15

B6129 WT and Cav1-KO mice (n = 15 per genotype) were infected with *H. pylori* SS1 for 11 months. Histopathological scores of gastritis were evaluated in H&E stainings of sections from paraffin-embedded stomach tissue. Score 0 = no, 1+ = mild, 2+ = moderate, 3+ = severe gastritis. Fisher Exact p = 0.001 (score 0 *versus* score 2+); Fisher Exact p = 0.002 (score 0 *versus* scores 1+,2+).

### Cav1-deficiency promotes recruitment of macrophages into the infected gastric mucosa

**Figure 2:**
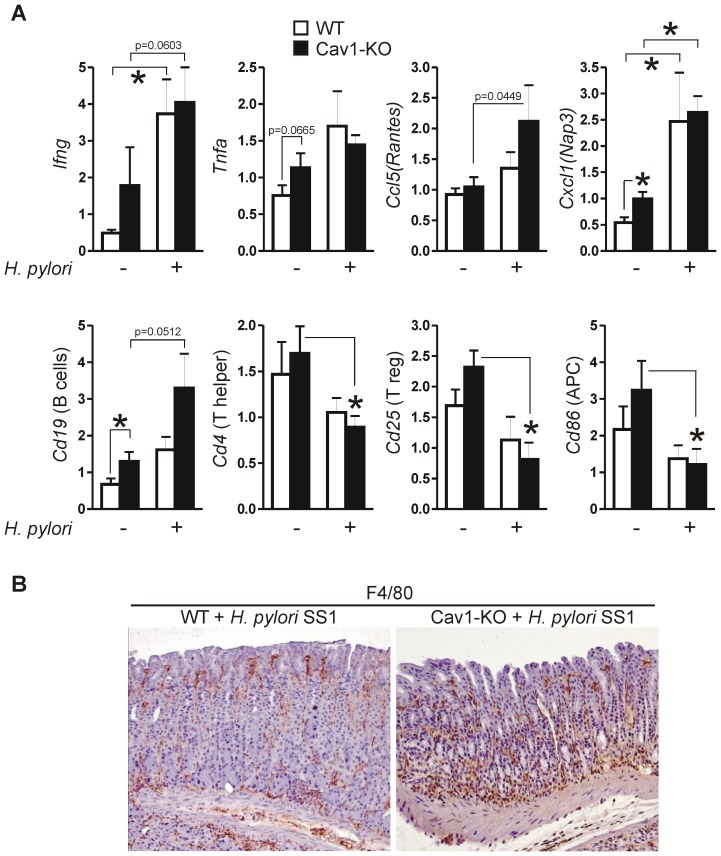
Loss of Cav1 promotes recruitment of macrophages to stomachs infected with *H. pylori* SS1. (A) Differential expression of mRNAs in mouse gastric tissue upon an 11-month infection with *H. pylori* strain SS1. CT-values from RT-qPCRs on total RNA extracted from resected stomachs were normalized to beta-2-microglobulin (b2M) and are presented as mean ± S.E. (n = 15 per group); *p<0.05 as indicated by brackets and asterisks. Changes in mRNA levels were dependent on *H. pylori*-infection or genotype, respectively. (B) Cav1-KO mice infected with *H. pylori* SS1 for 11 month had pronounced infiltration of intramucosal macrophages. Immunohistochemistry (IHC) of F4/80-positive mononuclear cells. Representative images are shown.

To assess the identity of immune cells which contribute to *H. pylori*-related inflammation in Cav1-KO mice, RT-qPCR analysis of selected cytokines, surface markers and chemokines was performed (**Fig. 2A**). Consistent with the observed inflammation, *H. pylori* SS1 induced expression of TNFalpha and IFNgamma in the gastric mucosa of both WT and KO mice. In addition, we stated an increased mRNA expression of CD19 (B-cells) (1.6±0.3 WT *versus* 3.3±0.9 KO; p = 0.0512; n = 15 per genotype) and RANTES (CCL5) (1.3±0.2 WT *versus* 2.1±0.6 KO; p = 0.0449; n = 15 per genotype) in gastric tissue of *H. pylori*-infected Cav1-KO mice compared with infected WT mice. In contrast, mRNA levels of CD4 (T-helper cells), CD25 (T-regulatory cells) and CD86 (antigen-presenting cells) were suppressed by *H. pylori* independently of the Cav1 status. Immunohistochemistry (IHC) detected a marked increase of intramucosal F4/80-positive macrophages in gastric tissue of infected Cav1-KO mice compared with WT littermates (**Fig. 2B**). CD3-positive lymphocytes were located around and within intramucosal follicles (data not shown).

Similar results were obtained from experiments introducing rapid gastric injury in mice by injection of indomethacin [30] (**Fig.S1**). Consistent with the enhanced tissue damage in Cav1-KO stomachs (*p = 0.0161, WT *versus* KO, n = 9 per genotype), characterized by inflammation, erosion and ulceration, Cav1-deficient mice also expressed higher amounts of mRNAs encoding for the ulcer healing proteins trefoil factor-2 (TFF2) (0.8±0.3 WT *versus* 2.3±0.4 KO; *p = 0.0048; n = 9 per genotype) and peroxisome proliferator-activated receptor-gamma (PPARg) (0.6±0.2 WT *versus* 2.5±0.5 KO; *p = 0.0008; n = 9 per genotype). In sum, these data indicated that loss of Cav1 enhances the susceptibility of mice to gastric inflammation and tissue damage.

### Cav1 neither alters adhesion of *H. pylori* strains to nor survival of human GC cells

**Figure 3:**
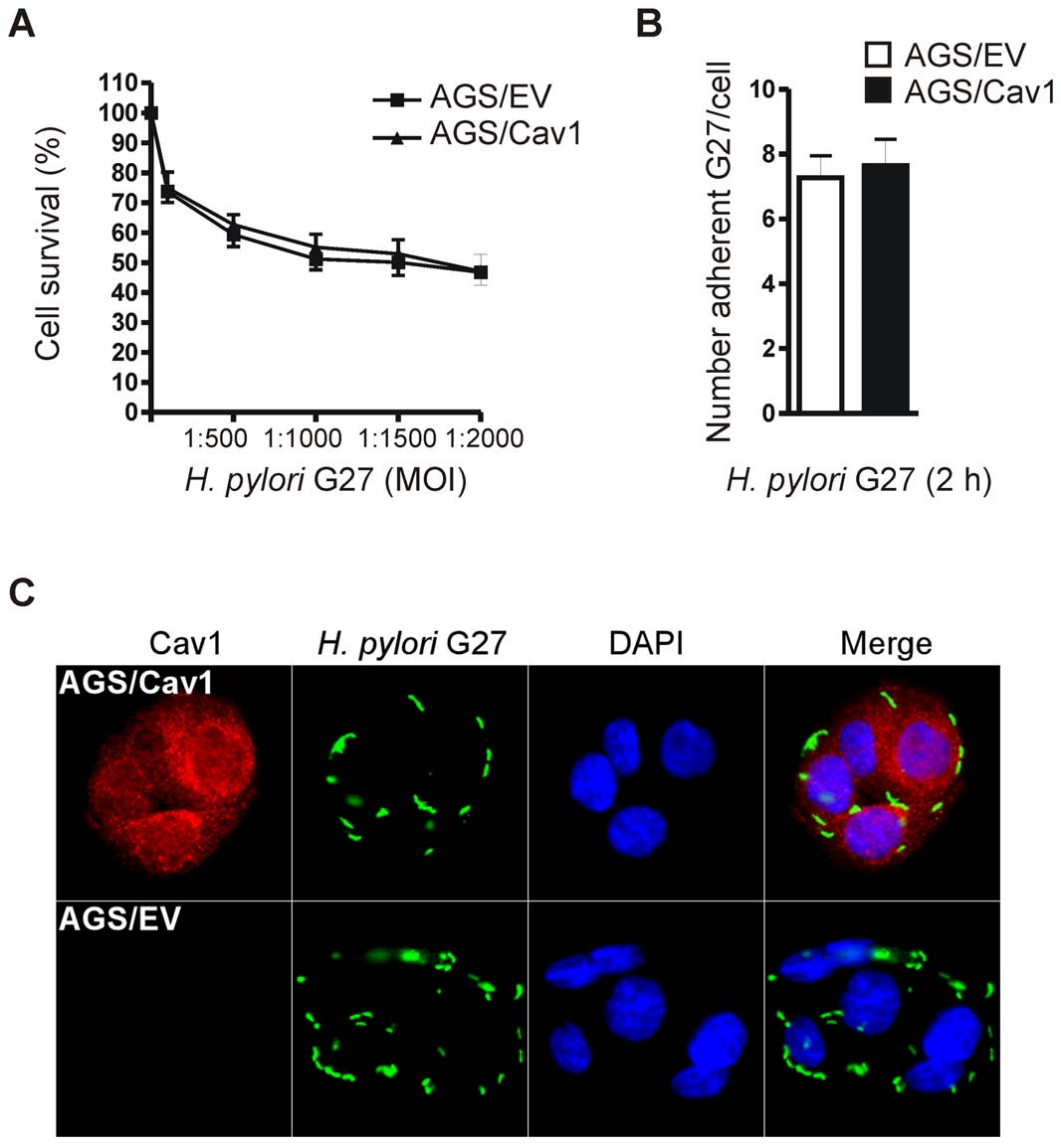
Cav1 has no effect on adhesion of *H. pylori* to or on viability of human GC cells. (A) Proliferation. AGS/Cav1 and AGS/EV cells (“AGS clones”) were infected with the cell-adapted CagA-delivery proficient *H. pylori* strain G27 strain for 48 h at MOI ratios of 1∶100 to 1∶2000. O.D. values from colorimetric MTT assays were calculated as % survival ± S.E. (n = 3); n.s. Cav1 *versus* EV. (B–C) Adhesion. AGS clones were infected with G27 (MOI = 10) for 30 min, washed, and additionally incubated in fresh medium for 2 h before staining for immunofluorescence microscopy; green = *H. pylori*, red = Cav1, blue = nuclei. Magnification ×630. Representative images (C) are shown together with a quantitative analysis (B). Adherent bacteria per Cav1-positive and Cav1-negative cells were counted (>10 cells per field, total of 5 fields, n = 3 experiments) and presented as mean ± S.E.; n.s. Cav1 *versus* EV.

To assess the function of Cav1 during *H. pylori* infection *in vitro*, the human gastric epithelial cell line AGS was used which had been stably transfected with Cav1 expression plasmid (AGS/Cav1) or empty vector (AGS/EV) [37]. First, we examined whether Cav1 influences cell survival upon *H. pylori* infection (**Fig. 3A**). AGS clones with and without Cav1 were infected for 48 h with the cell-adapted CagA-delivery competent *H. pylori* strain G27 at different multiplicities of infection (MOI) ranging from 1∶100 to 1∶2000. Colorimetric MTT assays revealed that Cav1 had no effect on overall survival of AGS cells upon *H. pylori* infection. Similar results were obtained with CagA-delivery incompetent *H. pylori* SS1 and by Western blot (WB) analysis detecting the expression and phosphorylation of survival kinases (AKT/PKB, ERK1/2, p38MAPK) (data not shown). Since both *H. pylori* and Cav1 interact within lipid rafts, we asked whether adhesion of bacteria to cells depends on the presence of Cav1. AGS/Cav1 and AGS/EV cells were infected (MOI = 10) with G27 (**Fig. 3B,C**) or SS1 (data not shown) bacteria for 30 min, followed by washing and subsequent incubation in fresh medium for 2 h. Thereafter, cells were stained for immunofluorescence microscopy, and the number of bacteria which adhered to the Cav1-expressing or empty vector-transfected cells were counted (**Fig. 3B,C**). No differences in adhesion were observed between AGS/Cav1 and AGS/EV cells, suggesting that Cav1 does not influence adhesion of *H. pylori* bacteria to host cells.

### Cav1 protects human GC cells against CagA-induced rearrangement of the cytoskeleton

**Figure 4:**
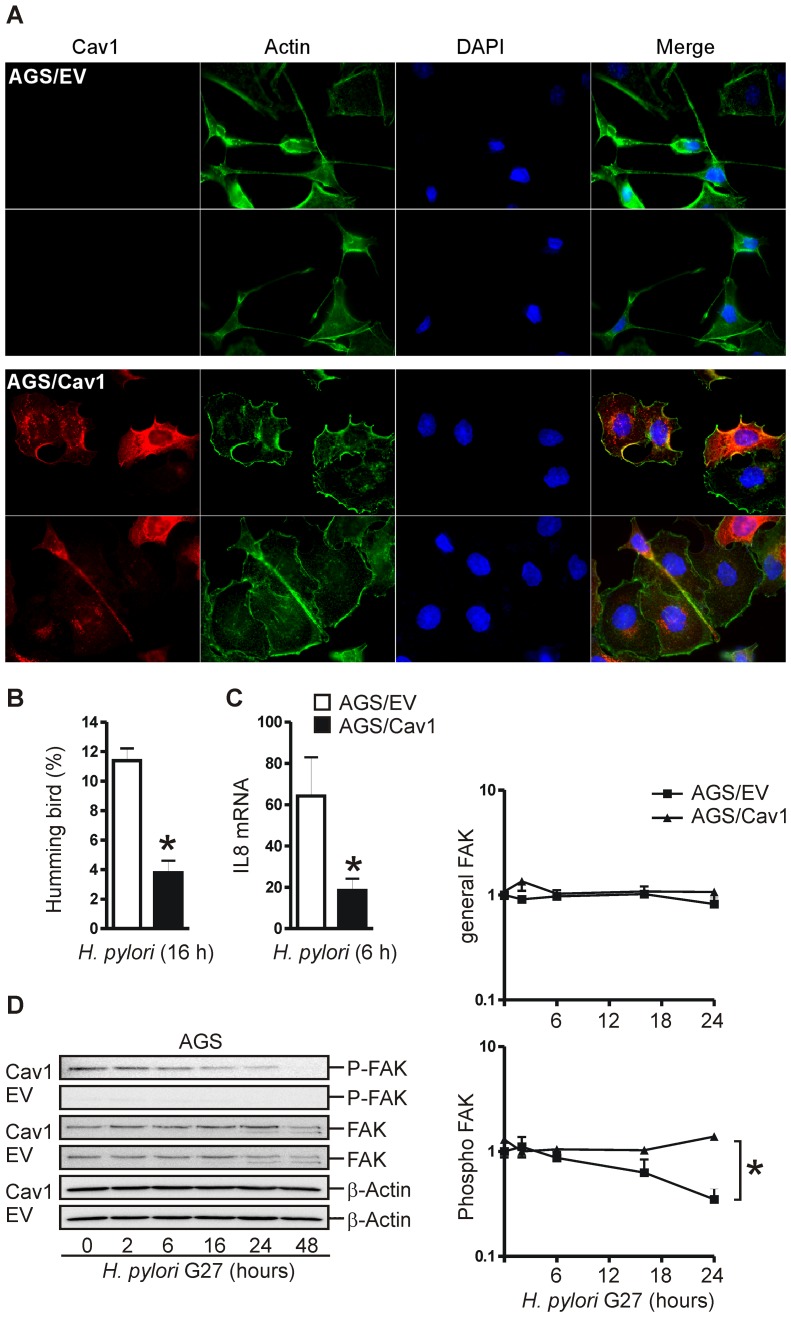
Cav1 protects human GC cells against CagA-induced cytoskeletal stress. (A–B) Cav1 inhibits CagA-induced cytoskeletal responses (“humming bird”). AGS clones were infected with CagA-delivery proficient *H. pylori* G27 (MOI = 100) for 16 h and stained for immunofluorescence microscopy; green = actin (phalloidin), red = Cav1, blue = nuclei. Magnification ×630. Note the needle-like projections of AGS/EV cells compared to spread-out epithelial AGS/Cav1 cells. Representative images (A) are shown above the quantitative analysis (B). Morphologies of all cells (>10 cells/per field; total of 10 fields, n = 3 experiments) were counted and calculated in % “humming bird” ± S.E. compared to the total cell number; *p = 1.1×10^−8^ AGS/Cav1 *versus* AGS/EV. (C) Cav1 inhibits *H. pylori*-induced production of IL8 mRNA. Cells were treated as in (A) for 6 h. CT-values from RT-qPCRs were normalized to b2M and presented as mean ± S.E. (n = 3 per clone); *p = 0.0176 AGS/Cav1 *versus* AGS/EV. (D) AGS/Cav1 cells have enhanced protein levels of phosphorylated (abbrev. P-) focal adhesion kinase (FAK), which promotes cell adhesion and spreading (see also **Figure S2**), thereby opposing CagA-induced cytoskeletal stress. Representative Western Blots (WB) from total cell lysates (left) are show together with a quantitative summary (right). O.D. values from bands in gels were calculated as -fold ± S.E. (n = 3) compared to time point 0 (uninfected cells); * p = 0.0012 AGS/Cav1 *versus* AGS/EV.

The formation of needle-like projections (“humming bird”) is a typical morphological phenotype of AGS cells in response to infection with CagA-delivery proficient *H. pylori* strains and translocation of CagA into the cytosol [47]. To examine the role of Cav1 in this stress-induced rearrangement of the actin cytoskeleton, AGS/Cav1 and AGS/EV were infected for 16 h with *H. pylori* G27 *wt* or the isogenic mutant *Delta cagA* (MOI = 100). Infected cells were stained as described above, and the numbers of elongated AGS cells were determined (**Fig. 4A,B**). Cav1-deficient AGS cells showed considerably more elongated morphologies than Cav1-expressing cells (11±0.8% AGS/EV *versus* 4±0.8% AGS/Cav1; *p = 1.1×10^−8^; n = 3 per clone). As expected, no “humming bird” phenotype was obtained in cells infected with the CagA-delivery deficient SS1 or the CagA-deletion mutant G27 *Delta cagA* strains which are both unable to inject functional CagA protein into the host cells (data not shown). AGS/EV cells also produced more IL8 mRNA upon *H. pylori* G27 infection than AGS/Cav1 cells (64±19 EV *versus* 19±6 Cav1; *p = 0.0176; n = 3 per clone) (**Fig. 4C**). These data indicated that Cav1 protects against CagA-related cell stress.

In support of these findings, cell adhesion and wound closure rates were more pronounced in AGS/Cav1 compared with AGS/EV cells (**Fig.S2**). Consistent with its function as a target protein of CagA and component of focal adhesions [48], WB analyses (**Fig. 4D**) also detected higher levels (0.4±0.1 AGS/EV *versus* 1.4±0.1 AGS/Cav1, *p = 0.0012; n = 3 per clone) of phosphorylated focal adhesion kinase (FAK) in Cav1-expressing cells infected with *H. pylori* G27. These data corroborated that AGS/Cav1 cells infected with CagA-delivery competent *H. pylori* maintained their spread-out epithelial shape as compared with the stressed elongated phenotype of Cav1/EV cells.

### CagA-delivery competent *H. pylori* G27 triggers binding of p120RhoGAP/DLC1 to Cav1 in human GC cells

**Figure 5:**
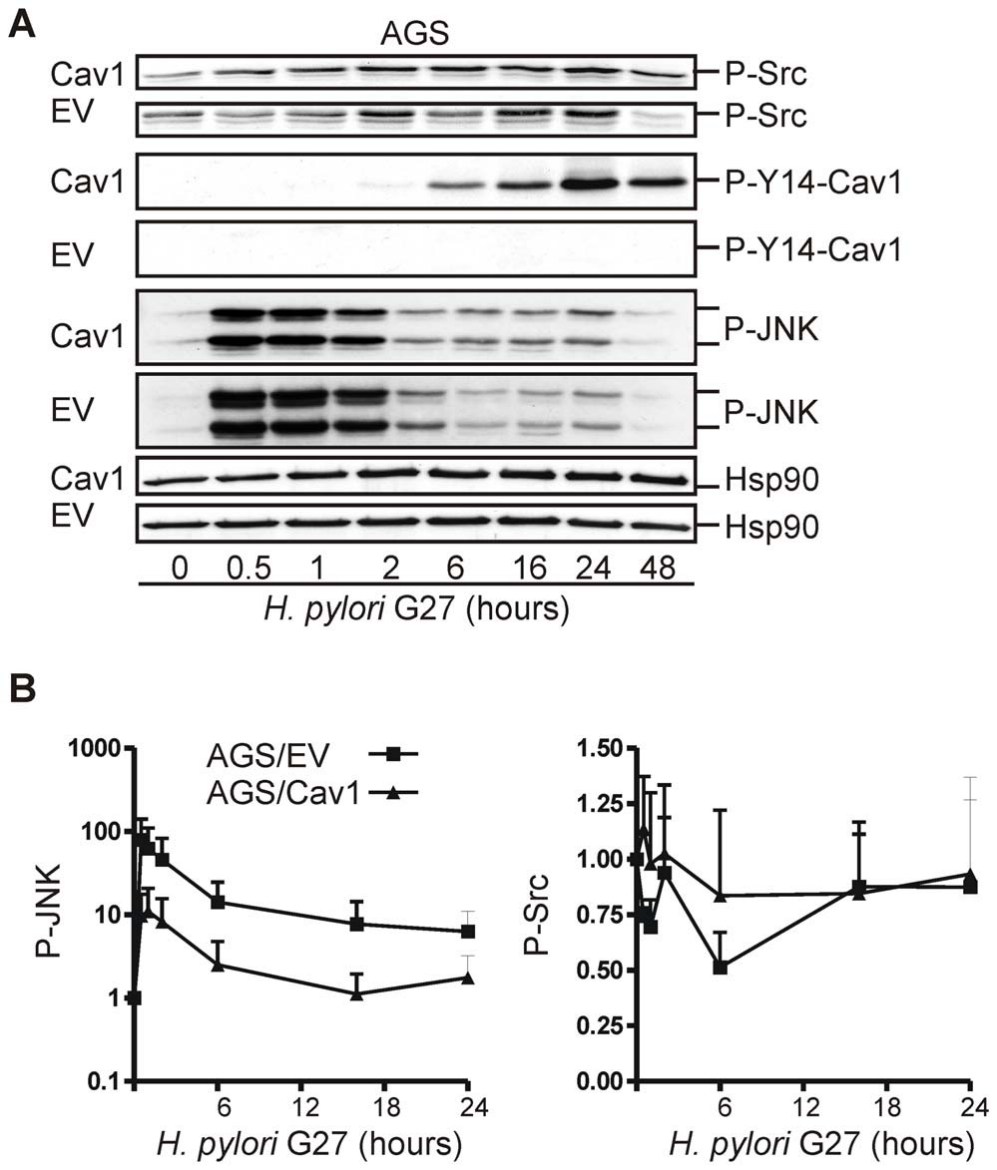
CagA evokes phosphorylation of Cav1 and Src independently of Cav1. (A–B) AGS clones were infected with CagA-delivery proficient *H. pylori* G27 (MOI = 100) for 30 min to 48 h, and cell lysates were analysed by WB. Representative WBs (A) are shown together with the quantitative summary (B). O.D. values from bands in gels were calculated as -fold ± S.E. (n = 3) compared to time point 0 (uninfected cells). n.s. AGS/Cav1 *versus* AGS/EV.

Cav1 has been shown to be phosphorylated by cytosolic tyrosine kinases (Src, Abl) at tyrosine 14 [49], and phosphorylated Cav1 and Src both activate the small GTPases Rho/Rac/Cdc42 which regulate cytoskeletal functions [13], [50]. To identify the underlying molecular mechanism how Cav1 protects against CagA-related cell stress, we assessed the signalling pathways initiated by CagA-delivery proficient *H. pylori* G27. Infection of AGS cells evoked a rapid phosphorylation of Cav1 in AGS/Cav1 cells and of Src in both AGS/Cav1 and AGS/EV cells. This result indicated that Cav1 acts downstream of CagA-dependent Src activation but upstream of the activation of the small GTPases (**Fig. 5A,B**). Consistent with this conclusion, protein levels of phosphorylated JNK, which resides below of Src, were higher in AGS/EV cells compared with AGS/Cav1 cells.

**Figure 6:**
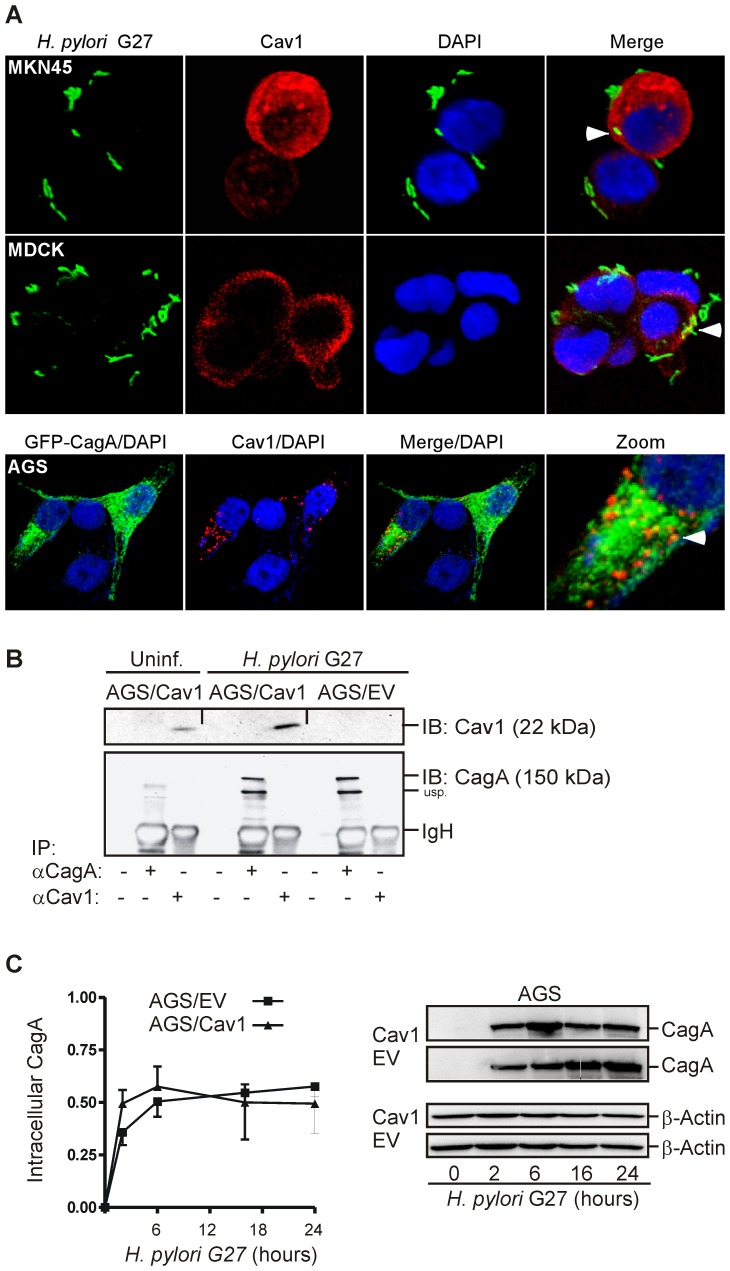
Cav1 does not interact with CagA but inhibits downstream effects of CagA. (A) Cav1 does not quantitatively colocalize with *H. pylori* or CagA. AGS/Cav1, MDCK and MKN45 cells were infected with CagA-delivery proficient *H. pylori* G27 (MOI = 10) or were transiently transfected with GFP-CagA expression plasmid, followed by staining for confocal immunofluorescence microscopy; green = *H. pylori*/GFP-CagA, red = Cav1, blue = nuclei. Magnification ×630. Occasional colocalizations appear in yellow. (B) Cav1 does not directly interact with CagA. Cells were infected as in (A) for 16 h (MOI = 100), and cell lysates were incubated with rabbit polyclonal CagA and Cav1 antisera. Precipitated proteins were detected by WB. Representative CoIP experiments are shown. (C) Gentamycin protection assay. Cav1 has no impact on injection of CagA into the host cell. AGS clones were infected with *H. pylori* G27 (MOI = 500) for 2 to 24 h, extensively washed with antibiotics, and intracellular CagA was determined by WB. Representative results are shown next to the quantitative analyses. O.D. values from bands in gels were calculated as mean ± S.E. (n = 3 independent experiments); n.s. AGS/Cav1 *versus* AGS/EV.

We were unable to detect a direct interaction or quantitative colocalization of CagA protein or *H. pylori* G27 bacteria with Cav1 in CoIP or immunofluorescence experiments (**Fig. 6A,B**). Gentamycin protection assays revealed that the total amount of injected intracellular CagA was also independent of Cav1's presence (**Fig. 6C**). Thus, Cav1 neither inhibited adhesion of *H. pylori* bacteria to nor injection of CagA into the host cell, but rather reduced the down-stream effects of CagA on intracellular signalling.

**Figure 7:**
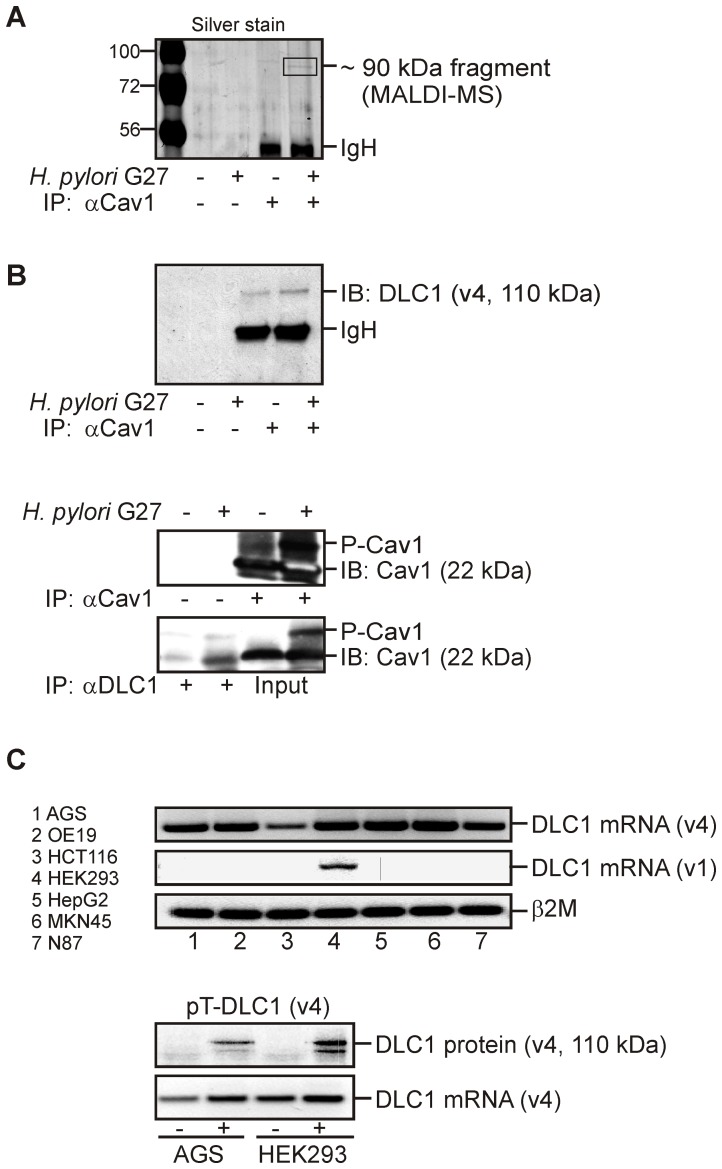
Cav1 binds p120RhoGAP/DLC1 upon infection of human GC cells with *H. pylori* G27. (A) Cav1 interacts with p120RhoGAP/DLC1, a tumor suppressor and inhibitor of small GTPases associated with focal adhesions and caveolae/lipid rafts. AGS/Cav1 cells were infected with CagA-delivery competent *H. pylori* G27 (MOI = 100) for 16 h. Total cell lysates were subjected to CoIP with rabbit polyclonal Cav1-antiserum. Gels were silver stained, and peptides within a band (∼95 kDa) enriched in infected cells were analysed by MALDI-MS (see also **Table S2**). (B) Validation of MALDI-MS. Cells were infected as (A), and total cell lysates were subjected to CoIP using antibodies against Cav1 and DLC1, respectively. Representative WBs show the ∼110 kDa DLC1 protein variant 4 (DLC1v4). (C) DLC1 mRNA and protein expression. Top panel: RT-PCR gels detecting DLC1 mRNA variant 1 (full length according to [39]) in HEK293 cells, whereas DLC1v4 (short form according to [39]) was expressed in all human cancer cell lines tested. Bottom panel: Cells were transiently transfected with the expression vector pTarget-DLC1v4 (pT-DLC1v4). Representative RT-PCR and WB gels visualizing the transfected DLC1v4 mRNA and the ∼110 kDa DLC1v4 protein are shown.

To identify a candidate protein which confers protection against CagA in a Cav1-dependent manner, a protein interaction screen based on MALDI-MS was performed (**Fig. 7A**). AGS/Cav1 cells were infected for 16 h with *H. pylori* G27 (MOI = 100) followed by lysis of the cells at room temperature in MES-buffered 1% (v/v) Triton-X100. Protein bands precipitated by Cav1 antiserum were visualized by silver staining, and peptides were identified by MALDI-MS as published previously [29]. A protein fragment of ∼95 kDa contained peptides corresponding to variant 4 of p120 Rho GTPase-activating protein/deleted in liver cancer-1 (p120RhoGAP/DLC1) [51], [52], a tumor suppressor associated with focal adhesions and caveolae/lipid rafts [53]. DLC1 variant 4 (DLC1v4) has a predicted size of ∼110 kDa and was enriched in samples from cells that had been infected with *H. pylori* G27 compared to uninfected cells (**Table S2**). These results were confirmed by CoIP of Cav1 and endogenous DLC1 protein in AGS/Cav1 cells (**Fig. 7B**), indicating that *H. pylori* G27 evoked a specific recruitment of DLC1 to Cav1 in infected human gastric epithelial cells.

**Figure 8:**
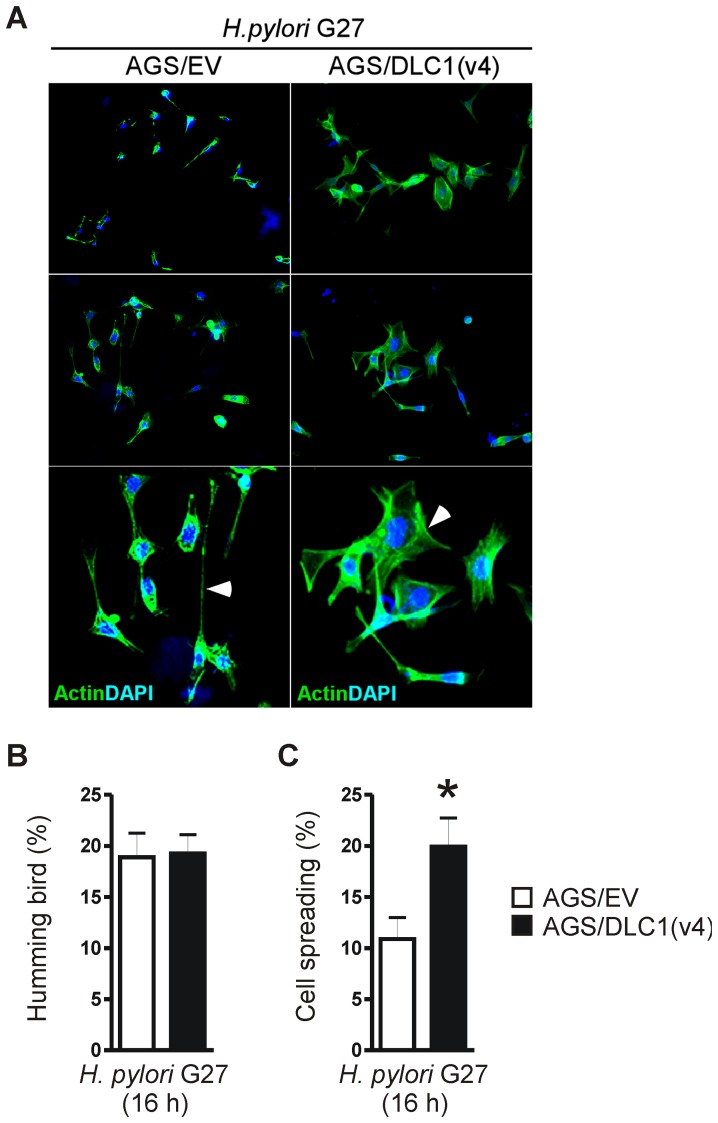
DLC1 rescues human GC cells from CagA-related adhesion defects. (A) AGS/EV cells were transiently transfected with pT-DLC1v4 plasmid for 24 h, followed by infection with CagA-delivery competent *H. pylori* G27 for additional 16 h and staining for immunofluorescence microscopy; green = actin (phalloidin), blue = nuclei. Magnification ×630. (B–C) Representative immunofluorescence images (A) are presented together with quantitative analyses of “humming bird” (B) and cell spreading (C) morphologies. Cell phenotypes were counted for at least 10 cells per field (total of 10 fields) and calculated as % ± S.E. (n = 3 experiments) of total cells; p = 0.0067 AGS/EV *versus* AGS/DLC1.

This result prompted us to amplify the cDNA of variant 4 of human DLC1 [39] from human hepatoma HepG2 cells (**Fig. 7C**). The cDNA was inserted into the expression vector pTarget (pT-DLC1v4) followed by transient transfection into parental AGS or HEK293 cells for 24 h. WB analyses detected expression of a ∼110 kDa protein, consistent with the predicted size of DLC1v4 [39]. Transiently transfected AGS cells were then infected with *H. pylori* G27 (MOI = 100) for additional 16 h. Immunofluorescence staining revealed that DLC1 *per se* did not inhibit formation of the CagA-induced “humming bird” phenotype (19±2% AGS/DLC1 *versus* 19±2% AGS/EV; n = 3 per clone) compared with empty vector-transfected cells (**Fig. 8A,B**). Instead, DLC1 promoted cell spreading (20±3% AGS/DLC1 *versus* 11±2% AGS/EV; *p = 0.0067; n = 3 per clone) consistent with its role in regulation of focal adhesions [54], [55], [56] (**Fig. 8A,C**). Pull-down assays which detected the activity of the small GTPases Rho/Rac/Cdc42 corroborated previous findings [56], [57], [58] that the CagA-proficient *H. pylori* G27 strain was a weak activator of these GTPases (data not shown). In sum, this data proposed that Cav1 inhibited CagA-induced cytoskeletal changes through alterations in the assembly or disassembly of focal adhesions via FAK rather than via the small GTPase pathways.

### *H. pylori* strains inhibit expression of Cav1 mRNA *in vivo* and *in vitro* independently of CagA

**Figure 9:**
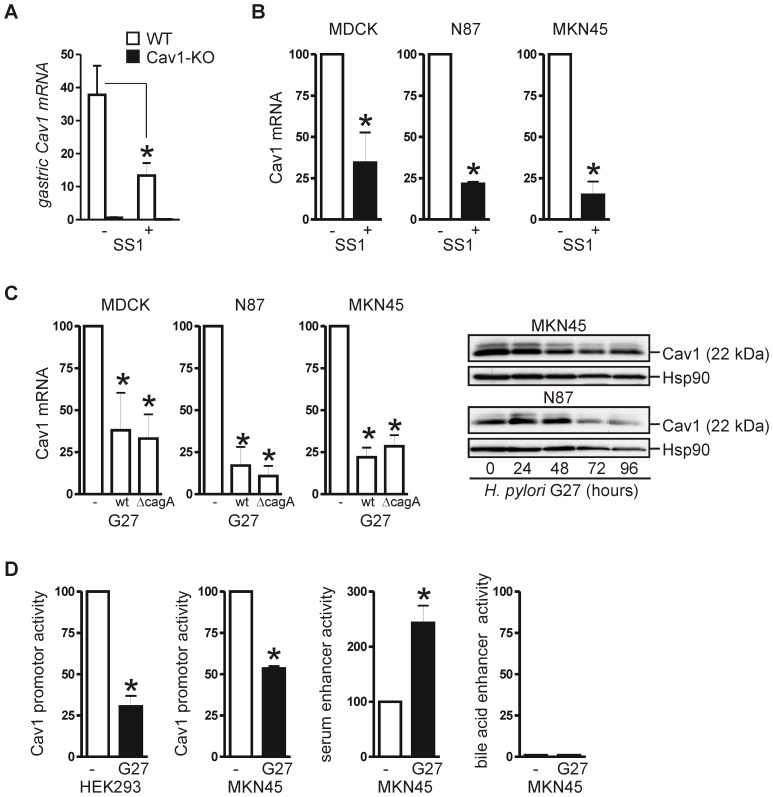
*H. pylori* strains down-regulate Cav1 gene expression *in vitro* and *in vivo* independently of CagA. (A) Reduced expression of mouse C*av1* mRNA after an 11-month infection with CagA-delivery defective *H. pylori* strain SS1. CT-values from RT-qPCRs on total RNA extracted from resected stomachs were normalized to b2M and presented as mean ± S.E. (n = 15 per group); *p = 0.0081 KO *versus* WT. (B–C) CagA-proficient G27 and CagA-delivery incompetent SS1 *H. pylori* strains both down-regulate Cav1 mRNA and protein expression in human GC cell lines independently of CagA. MDCK, N87 and MKN45 cells were infected with the respective *H. pylori* strain (MOI = 100) for the indicated times. Left: CT-values from RT-qPCRs on total RNA (3 days upon infection) were normalized to b2M and calculated as % ± S.E. (n = 3 per cell lines); (B) *p = 0.0043 (MDCK), 0.0001 (N87) and 0.0345 (MKN45), (C) *p<0.05 for all three cell lines; *H. pylori versus* mock. Right: Representative WBs are shown. (D) *H. pylori* inhibits Cav1 promoter activity. HEK293 and MKN45 cells were transiently transfected for 24 h with pGL3-*CAV1*p, SeRE or BSEP firefly luciferase reporter plasmids together with renilla control plasmid, followed by infection with *H. pylori* G27 (MOI = 100) for additional 16 h. Firefly luciferase activity was normalized to renilla and is presented as % ± S.E. (n = 3); *p = 0.002 (Cav1) for HEK293, 2.7×10^−6^ (Cav1) and 0.0052 (SeRE) for MKN45, *H. pylori versus* mock.

We showed previously that Cav1 is frequently down-regulated in human GC [37]. We therefore asked whether *H. pylori* infection contributes to repression of the Cav1 gene. RT-qPCR analyses of total RNA isolated from stomach tissue of uninfected mice and mice infected with CagA-delivery incompetent *H. pylori* SS1 (for 11 month) were performed. Infected WT mice showed a significantly reduced expression of *Cav1* mRNA (13±4 WT+*H. pylori versus* 38±9 WT mock;*p = 0.0081; n = 15 per group) as compared to the uninfected WT mice (**Fig. 9A**).

Similar results were obtained from *in vitro* studies. Two different human GC cell lines with endogenous Cav1 expression, N87 and MKN45, and MDCK cells were infected for 3 days with CagA-delivery incompetent SS1 (**Fig. 9B**) or CagA-proficient G27 (**Fig. 9C**) *H. pylori* strains. In all cell lines, a robust reduction of Cav1 mRNA expression (by 62 to 85%; *H. pylori versus* mock; *p = 0.0001 to 0.0043; n = 3 per cell line) was observed compared with uninfected cells. Similar results were obtained for Cav1 protein by WB (**Fig. 9C**).

The mouse-adapted *H. pylori* SS1 strain, which had been used for our *in vivo* infections, contains the *cagA* gene, expresses *cagA* mRNA (data not shown) but does not exert CagA protein-dependent effector functions [40], [59], whereas the cell-adapted G27 strain delivers active CagA [56] into the host cells. We therefore assessed whether Cav1 down-regulation is CagA-dependent or not. The same three cell lines were infected with *H. pylori* G27 *Delta cagA* (**Fig. 9C**) for three days. The CagA-deleted strain also decreased the amounts of Cav1 mRNA compared with uninfected cells (by 67 to 89%; *H. pylori versus* mock; *p = 6.1×10^−5^ to 0.0249; n = 3 per cell line), emphasizing that the repression of the Cav1 gene was CagA-independent *in vitro* and *in vivo*.

To determine whether the down-regulation of the Cav1 mRNA was caused by inhibition of the Cav1 promoter, reporter assays were performed (**Fig. 9D**). MKN45 cells were transfected with a luciferase reporter plasmid pGL3 containing the human proximal Cav1 promoter (pGL3-*CAV1*p) followed by a 16 h infection with CagA-proficient *H. pylori* G27 (MOI = 100). As a positive control served the pGL3-SeRE plasmid which harboured a CagA/stress-responsive serum-response element (SeRE) [60]. *H. pylori* G27 infection significantly reduced the activity of the Cav1 promoter (to 53±1% *H. pylori versus* mock; *p = 2.7×10^−6^ to 0.0052; n = 3) compared with uninfected cells. Similar results were obtained from HEK293 cells (**Fig. 9D**) and with CagA-delivery incompetent *H. pylori* SS1 (data not shown). In contrast, the activity of the SeRE was increased in *H. pylori* G27 infected MKN45 cells but not of an unrelated control promoter from the human bile salt export pump (BSEP) (**Fig. 9D**). This data confirmed that Cav1 gene expression is down-regulated at the transcriptional level independently of CagA.

### *H. pylori* strains activate nuclear SREBP1 to repress the human Cav1 promoter

**Figure 10:**
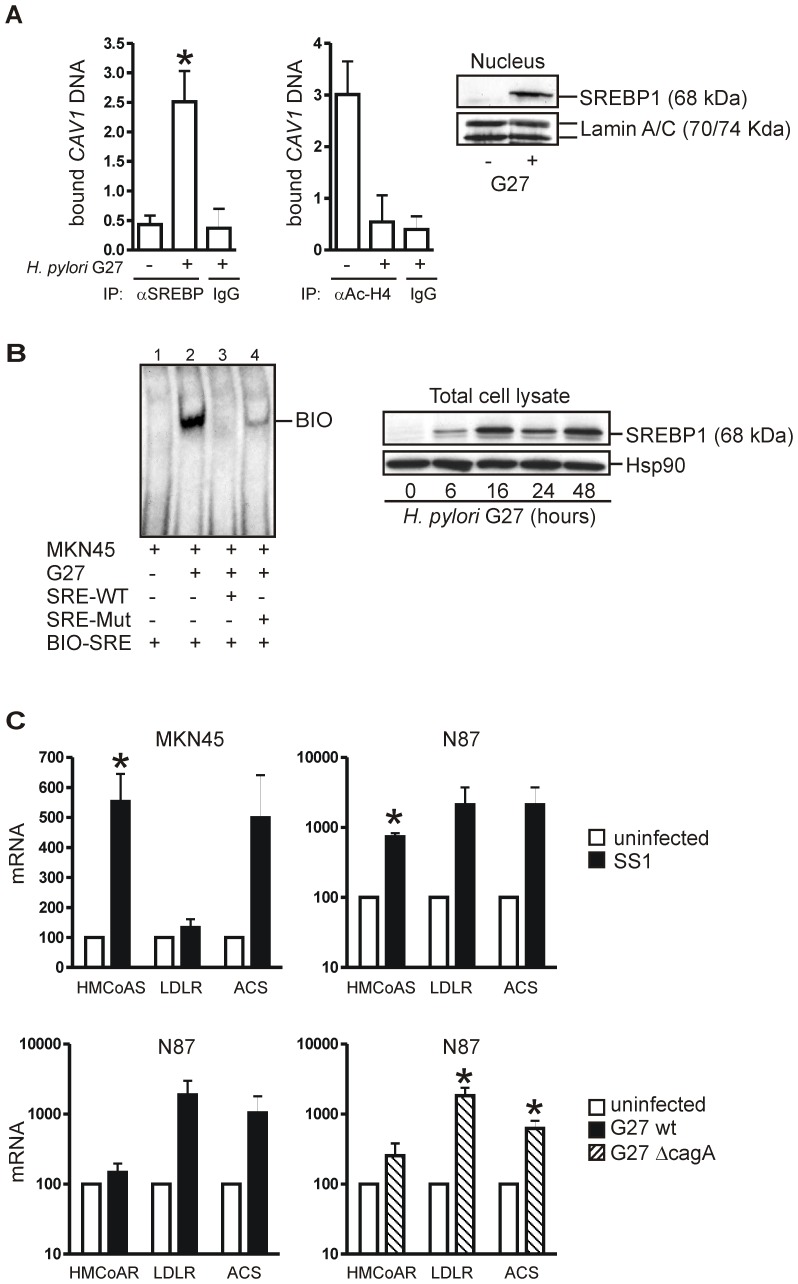
*H. pylori* strains down-regulate Cav1 promoter activity via activation of SREPB1. (A) *H. pylori* enhances binding of SREBP1 to one of the three sterol-responsive elements (SRE3) [24], [62] in the proximal human Cav1 promoter. ChIP: MKN45 cells were infected with *H. pylori* G27 (MOI = 100) for 16 h, and IP was performed in total cell lysates with rabbit polyclonal acetyl-H4-histone, SREBP1 or control IgG. CT-values from qPCR of IP-ed DNA were normalized to the CT-values of input DNA and were calculated as mean ± S.E. (n = 3) of protein-DNA complex pull-down by *H. pylori* compared to empty bead and uninfected controls; *p = 0.0093 for SREBP1 and p = 0.0478 for acetyl-H4-histone, *H. pylori versus* mock. Insert: Representative WBs of nuclear extracts showing mature 68 kDa SREBP1 in the nucleus of *H. pylori* G27-infected MKN45 cells. (B) EMSA: MKN45 cells were infected with *H. pylori* G27 for 16 h, and nuclear extracts were incubated with biotin (BIO)-labelled oligonucleotide (SRE3) [62] from the human Cav1 promoter and an excess of unlabelled competitor oligonucleotides (SRE3-WT = wild-type; SRE3-MUT = mutant). Insert: Representative WBs of whole cell lysates showing accumulation of active 68 kDa SREBP1 in *H. pylori* G27-infected MKN45 cells. (C) *H. pylori* also regulates mRNA expression of other cognate SREBP1 target genes. CT-values from RT-qPCRs on total RNA extracted from cells infected with CagA-delivery competent G27 *wt*, CagA-deleted G27 *Delta cagA* or CagA-delivery deficient SS1 *H. pylori* strains were normalized to b2M and presented as % ± S.E. (n = 15 per group); *p = 0.0137 to 0.0196, *H. pylori versus* mock.

Next, we were interested to identify the *H. pylori*-responsive repressor of the Cav1 gene. *H. pylori* lowers cholesterol levels in the host [26], and SREBP1 is activated by sterol deficiency to negatively regulate Cav1 gene transcription [24]. We therefore examined whether there is a higher binding rate of active nuclear 68 kDa SREBP1 to the sterol-responsive elements (SREs) of the proximal human Cav1 promoter upon *H. pylori* infection. MKN45 cells were infected with *H. pylori* G27 for 24 h, and ChIP was performed using antisera against SREBP1 and H4-acetyl histone [45], a marker for transcriptionally active “open” chromatin. Immunoprecipitated DNA was amplified by an whole genome amplification approach [61] and used for genomic qPCR analysis (**Fig. 10A**). Upon infection, we observed an increased binding of SREBP1 to the sterol-responsive element-3 (SRE3) [24], [62] of the Cav1 promoter (2.5±0.5 *H. pylori versus* 0.4±0.2 mock;*p = 0.0093; n = 3). In contrast, the amount of H4-acetyl-histone protein at the SRE3 was reduced upon infection (0.5±0.5 *H. pylori versus* 3±0.6 mock;*p = 0.0478; n = 3). These results suggested that *H. pylori* inhibits transcription at this site by recruitment of SREBP1 as a repressor of the Cav1 gene.

We corroborated these results using EMSA (**Fig. 10B**) [45]. MKN45 cells were infected with *H. pylori* G27 (MOI = 100) for 24 h. We could detect binding of protein to the SRE3 oligonucleotide from the Cav1 promoter exclusively in nuclear extracts of infected cells. WB assays evinced that *H. pylori* G27 evoked accumulation of the active 68 kDa SREBP1 fragment in the nucleus. RT-qPCR analyses demonstrated that the expression of other *bona fide* SREBP1 target genes, which are positively regulated by SREBP1, was also affected by *H. pylori*. The mRNAs encoding 3-hydroxy-3-methyl-glutaryl-CoA synthase (HMGCOAS), HMGCOA reductase (HMGCOAR), low density lipoprotein receptor (LDLR) and acetyl-coenzyme A synthetase (ACS) were up-regulated by CagA-proficient G27 *wt*, CagA-deleted G27 *Delta cagA* and CagA-delivery deficient SS1 bacteria to a maximum of 21-fold (*H. pylori versus* mock; *p = 0.0137 to 0.0196; n = 3 per cell line) compared with uninfected cells (**Fig. 10C**). Conclusively, these data emphasized that the Cav1 promoter was inhibited by *H. pylori*-activated SREBP1 independently of CagA.

## Discussion

In this study, we describe a novel role for Cav1 in *H. pylori*-mediated gastritis and cell damage. Since many years, lipid rafts have been shown to mediate uptake of pathogens (virus, bacteria, parasites) and their toxins into host cells [8], [10], [11]. Internalization of the two major toxins of *H. pylori*, VacA and CagA, via clathrin-independent lipid raft-dependent endocytosis and the bacterial type IV secretion system have been thoroughly validated using *in vitro* systems, including the human gastric epithelial cell line AGS [4], [7]. However, the role of Cav1 in *H. pylori* infection *in vitro* and *in vivo* remained unknown.

Recent reports on Cav1-deficient mice revealed a general enhanced susceptibility to disease provoked by local or systemic infection through certain pathogens including bacteria (*Salmonella typhimurium, Pseudomonas aeruginosa*) or parasites (*Trypanosoma cruzi*) [12], [63], [64], [65], [66], [67], [68]. Cav1-KO mice succumb to systemic infection earlier and suffer from a more severe disease phenotype than WT littermates. This sensitivity is presumably caused by certain defects in either the innate or the adaptive immune system. Since the predominant cell types with Cav1 expression are macrophages [68] and endothelial cells [69], recruitment and maturation of leukocytes (e.g. of regulatory T-cells [70]) may be impaired in absence of Cav1. Loss of Cav1 in macrophages results in defective phagocytosis [68], [71] and altered release of nitric oxide [72] and pro-inflammatory cytokines (TNFalpha, IL1beta) [64], [65]. Bacterial lipopolysaccharide has been reported to up-regulate Cav1 expression in B-cells [23], and Cav1 was shown to be associated with molecules of the synapse between T-cells [21], [73] and antigen-presenting cells. Thus, systemic absence of Cav1 may impair immune responses to pathogens at multiple levels.

Consistent with these reports, we found that Cav1-deficient mice responded with an enhanced active chronic gastritis and tissue damage to infection with the CagA-delivery deficient *H. pylori* SS1 strain compared with WT littermates. This response was accompanied by loss of parietal cells and foveolar hyperplasia. A bias towards a T helper 1 immune response is expected to facilitate the elimination of *H. pylori* bacteria from infected stomachs, however, at the expense of a more severe gastritis in humans and mice [74], [75]. Consistent with this concept, we showed that Cav1-KO animals had a reduced bacterial burden but an augmented local infiltration of macrophages, marked formation of intramucosal lymph follicles and production of chemokines (e.g. RANTES/CCL5) in the infected gastric tissue. In line with the known immunomodulatory effects of *H. pylori* [76], [77], [78], the expression of CD-markers related to T helper (CD4/GATA4) and regulatory T cells (CD25/FOXP3) was suppressed upon an 11-month infection with *H. pylori* SS1, and this phenomenon was most pronounced in Cav1-KO mice. Although more detailed studies have to characterize the gastric milieu and the immune defects of Cav1-KO mice, one may conclude that loss of Cav1 enhances the susceptibility to pathogen-related disease by defects in the generation of a pathogen-directed adaptive T-cell immune response.

*H. pylori* has developed many strategies to evade the host's immune system [78]. It has been described that *H. pylori* is auxotrophic for cholesterol and extracts cholesterol from the host cell membrane to actively inhibit phagocytosis and modify the generation of adaptive T-cell responses [26], [79]. Depletion of host cell membranes from cholesterol inhibits CagA-dependent effects on cell elongation and IL8 production *in vitro* [27], [80]. These observations led us to the hypothesis that *H. pylori* may create a cholesterol-deficient microenvironment in/around infected cells which activates the cholesterol deficiency sensor SREBP1. Indeed, we demonstrated that SREBP1 was activated by *H. pylori* to down-regulate Cav1 gene expression *in vitro* and *in vivo*. This effect was independent of *H. pylori*'s major oncoprotein CagA, but was strain-specific, because it was not observed upon infection with other *Helicobacter* species such as *H. hepaticus* (unpublished observation). In contrast to the CagA and VacA delivery-competent G27 strain, SS1 bacteria fail to exert *bona fide* effector functions of CagA and VacA proteins within host cells [40], [59], [81]. This defect may be attributed to the type IV secretion system or toxin delivery. Thus, further studies are necessary to identify the factors which are responsible for activation of SREBP1 by SS1.

To explore one of the *in vitro* mechanisms how Cav1 protects against *H. pylori*-related cell damage, we demonstrated that Cav1 neither interfered with adhesion of *H. pylori* SS1 or G27 bacteria to human gastric epithelial cells nor with injection of G27-derived CagA protein into the cytosol. Instead, Cav1 inhibited the down-stream effects of intracellular CagA on the rearrangement of the actin cytoskeleton and on the production of IL8. This *in vitro* phenomenon of spike-like cell elongation (“humming bird”) is supposed to resemble, at least in part, an *in vivo* event which facilitates access of live *H. pylori* into favourable niches of the gastric epithelium for successful persistence of the microorganism within the host organ [78]. We found that Cav1 did not directly interact with CagA. Instead, Src kinase was phosphorylated on specific tyrosine residues upon infection with *H. pylori* G27 independently of Cav1. Cav1 altered the activation status of two kinases downstream of Src, it reduced phosphorylation of JNK but enhanced that of FAK, proposing that Cav1 blunts stress-related CagA-signalling downstream of active Src but promotes cell adhesion.

Mechanistically, we evinced that the CagA-proficient *H. pylori* G27 evoked the recruitment of p120RhoGAP/DLC1 to Cav1. DLC1 has been initially described as an inhibitor of small GTPases which localizes to focal adhesions and lipid raft/caveolae membrane microdomains [53], [54], [55], [82]. Cav1 may thus promote the function/activity of DLC1 as a tumor suppressor via direct interaction through a *bona fide* Cav1-binding motif [53] identified in DLC1. Unexpectedly, DLC1 did not inhibit the formation of fiber-like elongations in cells infected with CagA-delivery competent *H. pylori* G27 which have been attributed to activation of the small GTPases RhoA/Rac1/Cdc42 by CagA. This result may be explained by previous reports [56], [57], [58] showing that the G27 strain is only a weak activator of those GTPases. Instead, DLC1 promoted cell adhesion and spreading. This phenotype was presumably caused by changes in the assembly and/or disassembly of focal adhesions, since FAK is a direct target of *H. pylori*'s CagA [48] and, together with other components of focal adhesions, such as talin and tensins, directly interacts with DLC1 [53], [54], [55]. Conclusively, Cav1 seems to exert its protective effect against intracellular CagA effector functions on the actin cytoskeleton via DLC1. Cav1 did not require direct interaction with CagA to exert its pro-adhesive effects. Hence, the Cav1/DLC1 complex may also protect cells against CagA-delivery deficient *H. pylori* strains, including SS1, which have been used in our *in vivo* study. However, future experiments have to explore this assumption.

Cav1 is a ubiquitous adapter molecule in many immune receptor signalling pathways. One may thus speculate that *H. pylori* exploits down-regulation of Cav1 to subvert the host immune system or to enhance the signalling efficiency of its virulence factors in gastric epithelial cells. In case of clinical isolates from infected individuals, the pre-mouse Sydney strain-1 (PMSS1) or G27 [32], [77] those virulence factors may comprise CagA and VacA, in case of SS1, other yet unknown bacterial proteins could be involved.

Loss of Cav1, in its function as a tumor suppressor and inhibitor of growth factor receptor signalling which stabilizes cell-cell and cell-matrix contacts, is a hallmark of many human cancers including GC [19]. Absence of Cav1 in primary tumors promotes cell proliferation and enables clonal expansion [15], [19]. Similar to Cav1, DLC1 is a tumor suppressor silenced or deleted in many human cancer entities including GC, e.g. by gene methylation [39], [51]. Thus, down-regulation of Cav1 by *H. pylori* in stomach tissue *in vivo* may be part of an early molecular sequence of events in the transition of inflammation to GC also in humans.

We would like to emphasize that the aim of the current work was not the identification of novel virulence factors in the SS1 *H. pylori* strain, which are unknown since 15 years, but rather to clarify the role of Cav1 in *H. pylori*-induced gastric pathology by directly comparing the lesions obtained in the Cav1-KO mice to the results of other researchers who used SS1 *H. pylori* strain in other mouse genotypes or backgrounds (C57BL/6, B6129, BALB/c) [28], [33], [34], [83]. The Cav1-KO mice did not progress to gastric neoplasia with CagA-delivery incompetent SS1. We therefore additionally investigated the molecular mechanisms of Cav1 on CagA-delivery proficient G27 strain signalling in gastric epithelial cell lines, in order to strengthen the relevance of our findings to the situation in humans, where CagA-injection competent strains are associated with development of GC [1], [3], [5]. In the future, we shall infect Cav1-KO mice with PMSS1, an *H. pylori* strain which injects functionally active CagA protein into the host gastric epithelium [77], [84], [85].

Our study describes two different aspects of Cav1's role in stomach disease: (i) first, an *in vivo* protective role against *H. pylori*-induced inflammation which was independent of CagA/VacA, and (ii) second, an *in vitro* protective role against *H. pylori*-induced cytoskeletal rearrangement which was dependent on CagA and DLC1. These data comprise two separate aspects of *H. pylori* biology which are not easily reconciled. Nevertheless, our major objective was to present a first description of a beneficial role for Cav1 in *H. pylori*-related diseases, against gastritis *in vivo* and cytoskeletal stress *in vitro*, rather than to elaborate on the potential virulence mechanisms of SS1. To our knowledge, this novel role of Cav1 in *H. pylori* biology was previously unknown and may thus provide the initial basis for further detailed *in vivo* and *in vitro* studies

We have been well aware of the fact that *H. pylori* strain SS1 is incapable of exerting CagA and VacA-dependent effector functions [81], [83], [86], [87]. Over the years, a consensus has been reached that SS1 expresses CagA mRNA but does not inject functional CagA protein into the host cells via the type IV secretion system [40]. Similarly, SS1 is devoid of VacA-dependent vacuolating cytotoxicity and induction of IL8 [81]. Nevertheless, SS1 is still able to induce severe gastric pathology *in vivo* independently of these two important virulence factors, especially after chronic infection and persistent colonization [35], [59]. The SS1 virulence factors responsible for gastric inflammation, tissue damage and carcinogenesis have remained unknown since the introduction of the strain as a standardized reference model in 1997 [32].

Several alternative mechanisms of virulence have been described for SS1. For example, SS1 up-regulates matrix-metalloproteinases (MMPs) inducing inflammation and tissue damage independently of CagA [86]. Moreover, SS1 *per se* evokes mutations and genotoxic stress in mice, a pathology which may contribute to pre-neoplastic alterations in the gastric mucosa [33], [88]. Interestingly, the CagA-delivery competent PMSS1 strain promotes genotoxic stress as well independently of its common virulence factors CagA and VacA [89]. Instead, bacterial adhesion factors were necessary to achieve the mutagenic effect [89]. Nevertheless, SS1 bacteria deficient in certain adhesion factors still evoked severe gastric pathology *in vivo* (here in gerbils) [90], and the presence of CagA or VacA had no effect on the ability of HP strains to adhere or invade gastric epithelial cells *in vitro* [87]. Thus, the quest for pathogenic virulence mechanisms of SS1 is still ongoing.

These reports demonstrated, that single major virulence factors like CagA or VacA are not the exclusive responsible agents for the observed gastric histopathology induced by SS1, but rather a combination of so far unknown bacterial factors and last but not least the host immune response. Relating to the latter, *H. pylori*-induced changes in cholesterol content at host cell membranes and the well-described immunomodulating effect of *H. pylori* may be of higher importance for pathogenicity than the action of single cytotoxins/oncoproteins. Confirming this assumption, we showed that SS1 (CagA−/VacA−) and G27 (CagA+/VacA+) strains both down-regulated SREBP1-mediated Cav1 gene expression independently of CagA/VacA, constituting a potential novel pathogenic mechanism of *H. pylori* which acts independently of classical virulence factors.

## Supporting Information

Figure S1 **Cav1 protects against gastric injury** ***in vivo*** **.** (A–B) Cav1-KO mice are susceptible to indomethacin-mediated gastric injury. C57BL/6 WT and B6129 Cav1-KO mice received an i.p. injection of 35 mg/kg indomethacin (n = 9 per genotype) or NaCl (n = 3 per genotype) for 24 h, respectively. H&E stainings from paraffin sections of gastric tissue were evaluated for damage scores [30], [91]: 0+ no inflammation, 1+ superficial erosive gastritis, 2+ moderate discrete erosive gastritis, 3+ severe gastritis with elongated erosions and ulcerations. Representative H&E stainings (A) and damage scores (B) for individual mice are presented; *p = 0.0161 WT *versus* KO; magnifications 100×. (C) Cav1-KO mice express higher levels of gastric mRNAs (*Pparg, Tff2*) involved in mucosa regeneration. The CT-values from RT-qPCRs on total RNA extracted from resected stomach tissue were normalized to b2M and presented as mean ± S.E. (n = 9 per group); *p = 0.0008 for *Pparg* and 0.0048 for *Tff2*, WT *versus* KO. (D) Cav1-KO and WT mice produce similar levels of systemic pro-inflammatory cytokines. Serum cytokines were measured by ELISA and values were calculated as pg/ml ± S.E. (n = 9 per group).
(TIF)

Figure S2 **Cav1 promotes cell adhesion and wound closure** ***in vitro*** **.** (A) Cell adhesion. AGS/Cav1 and AGS/EV cells were seeded on tissue culture dishes for 6 h. The number of adherent cells was counted as mean ± S.E. (n = 3); *p = 0.0394, Cav1 *versus* EV. (B) Wound closure. Confluent AGS/Cav1 and AGS/EV cell monolayers were injured by a 5 mm wide scratch. Wound closure by cell migration was measured after 24 h in micrometer and calculated as % ± S.E. (n = 3); *p = 0.0061, Cav1 *versus* EV.
(TIF)

Table S1 **Oligonucleotides.** DNA-sequences of 5′-(forward) and 3′-(reverse) primers for detection of human, mouse and *H. pylori* genes by RT-qPCR are listed. Oligonucleotide sequences from the human *CAV1* gene promoter containing SREs were used for performance of EMSA and ChIP assays.
(DOC)

Table S2 **DLC1 peptides identified by MALDI-MS.** The amino acid sequence and the localization of peptides precipitated in CoIP experiments using Cav1 as a bait are presented. Peptides overlapped with both variant 1 and variant 4 of the human DLC1 protein. * Mascot total ion score by GPS Explorer 2 software; # Location of peptides identified by MALDI-MS in the coding sequence (CDS) of human DLC1 protein variants.
(DOC)
