# Supplementary figures and images for: Caveolin-1 Protects B6129 Mice against Helicobacter pylori Gastritis
Source: PLoS Pathog. 2013 Apr 11;9(4):e1003251. doi: 10.1371/journal.ppat.1003251 (PMC3623771; doi:10.1371/journal.ppat.1003251)

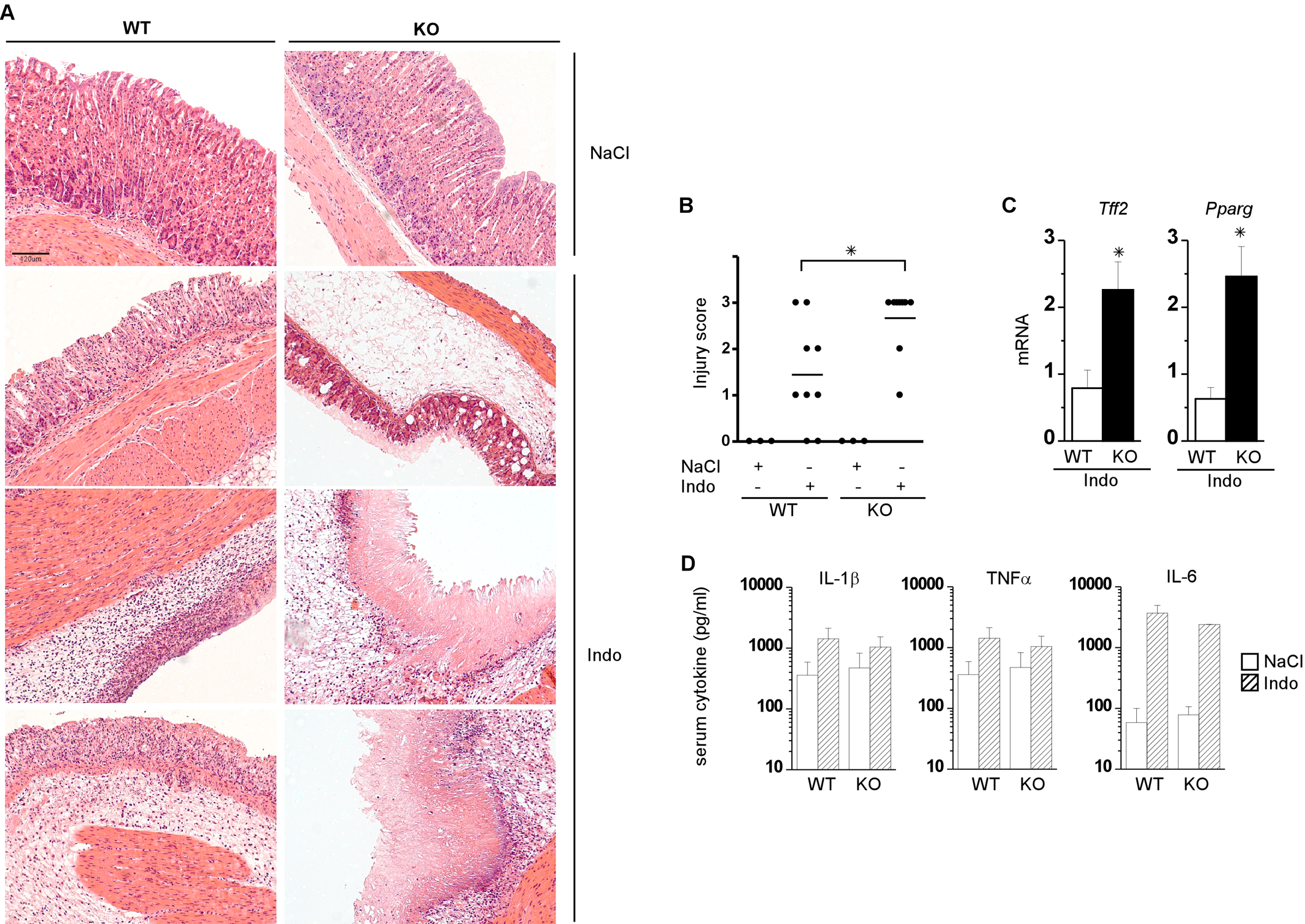

Supplement: Figure S1 — Cav1 protects against gastric injury in vivo . (A–B) Cav1-KO mice are susceptible to indomethacin-mediated gastric injury. C57BL/6 WT and B6129 Cav1-KO mice received an i.p. injection of 35 mg/kg indomethacin (n = 9 per genotype) or NaCl (n = 3 per genotype) for 24 h, respectively. H&E stainings from paraffin sections of gastric tissue were evaluated for damage scores [30], [91]: 0+ no inflammation, 1+ superficial erosive gastritis, 2+ moderate discrete erosive gastritis, 3+ severe gastritis with elongated erosions and ulcerations. Representative H&E stainings (A) and damage scores (B) for individual mice are presented; *p = 0.0161 WT versus KO; magnifications 100×. (C) Cav1-KO mice express higher levels of gastric mRNAs (Pparg, Tff2) involved in mucosa regeneration. The CT-values from RT-qPCRs on total RNA extracted from resected stomach tissue were normalized to b2M and presented as mean ± S.E. (n = 9 per group); *p = 0.0008 for Pparg and 0.0048 for Tff2, WT versus KO. (D) Cav1-KO and WT mice produce similar levels of systemic pro-inflammatory cytokines. Serum cytokines were measured by ELISA and values were calculated as pg/ml ± S.E. (n = 9 per group). (TIF) [file ppat.1003251.s001.tif]

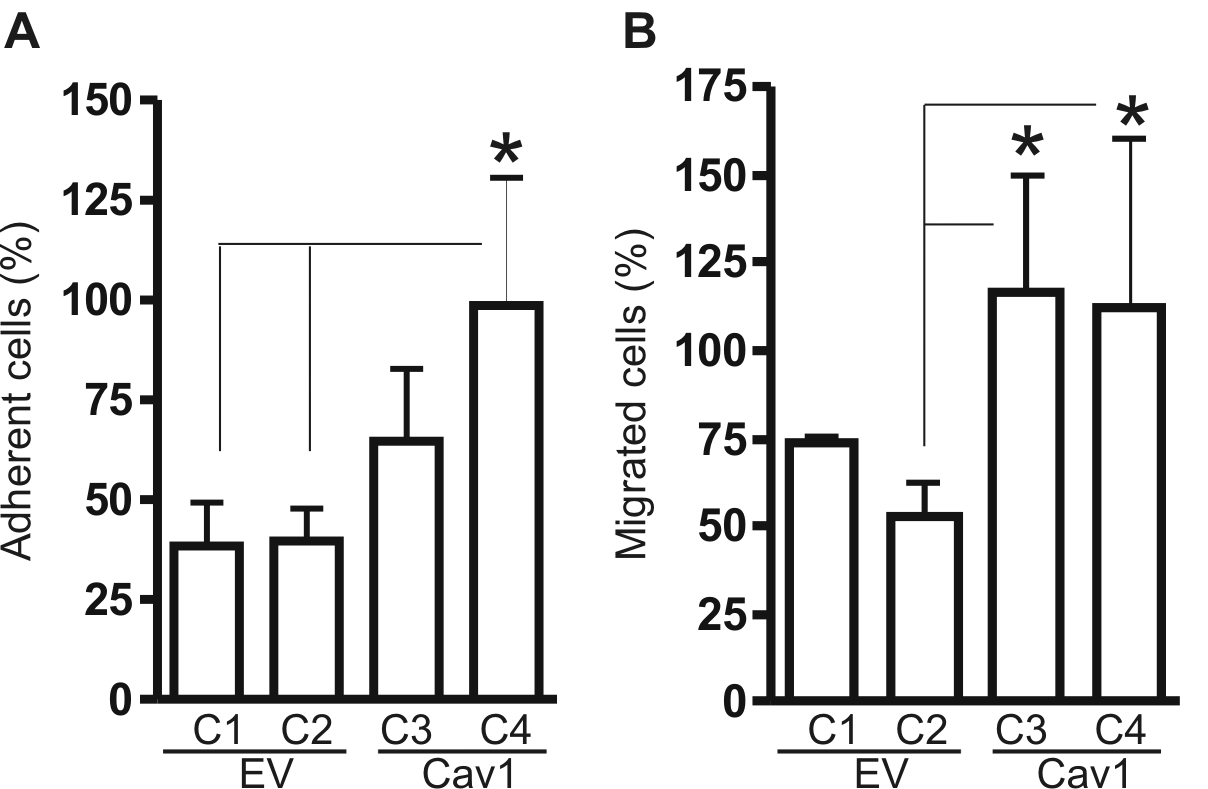

Supplement: Figure S2 — Cav1 promotes cell adhesion and wound closure in vitro . (A) Cell adhesion. AGS/Cav1 and AGS/EV cells were seeded on tissue culture dishes for 6 h. The number of adherent cells was counted as mean ± S.E. (n = 3); *p = 0.0394, Cav1 versus EV. (B) Wound closure. Confluent AGS/Cav1 and AGS/EV cell monolayers were injured by a 5 mm wide scratch. Wound closure by cell migration was measured after 24 h in micrometer and calculated as % ± S.E. (n = 3); *p = 0.0061, Cav1 versus EV. (TIF) [file ppat.1003251.s002.tif]
