# Supplementary material for: Caveolin-1 Protects B6129 Mice against Helicobacter pylori Gastritis
Source: PLoS Pathog. 2013 Apr 11;9(4):e1003251. doi: 10.1371/journal.ppat.1003251 (PMC3623771; doi:10.1371/journal.ppat.1003251)
Supplement: Table S1 — Oligonucleotides. DNA-sequences of 5′-(forward) and 3′-(reverse) primers for detection of human, mouse and H. pylori genes by RT-qPCR are listed. Oligonucleotide sequences from the human CAV1 gene promoter containing SREs were used for performance of EMSA and ChIP assays. (DOC) [file ppat.1003251.s003.doc]

**Table S1 Oligonucleotides.**

| **Primer** | **Forward (5’)** | **Reverse (3’)** |
| --- | --- | --- |
| **EMSA** | | |
| Human *SRE3* | AAGCACCCCAGCGCGGGACAACGTTCT | AGAACGTTGTCCCGCGCTGGGGTGCTT |
| Human *SRE3mut* | AATTAATTAATCGCGGGACAACGTTCT | AGAACGTTGTCCCGCGATTAATTAATT |
| **ChIP** | | |
| Human *CAV1-300* | gagatgatgcactgcgaaaa | gccaaaggtttgttctgctc |
| **RT-qPCR** | | |
| Mouse Cav1 | Agccgcgtctactccatcta | tctctttctgcgtgctgatg |
| Mouse b2M | ATGGGAAGCCGAACATACTG | CAGTCTCAGTGGGGGTGAAT |
| Mouse TNFa | ACGGCATGGATCTCAAAGAC | GTGGGTGAGGAGCACGTAGT |
| Mouse IFNg | GCGTCATTGAATCACACCTG | TGAGCTCATTGAATGCTTGG |
| Mouse IL-1b | GGGCCTCAAAGGAAAGAATC | TACCAGTTGGGGAACTCTGC |
| Mouse IL-6 | CCGGAGAGGAGACTTCACAG | TCCACGATTTCCCAGAGAAC |
| Mouse CD4 | AGGAAGTGAACCTGGTGGTG | CTCCTGCTTCAGGGTCAGTC |
| Mouse CD19 | GGACAGTGAACGTGGAGGAT | GGGCACATACAGGCTTTGTT |
| Mouse CD25 | AGAACACCACCGATTTCTGG | CTGTGGGTTGTGGGAAGTCT |
| Mouse CD86 | TCAGTGATCGCCAACTTCAG | TTAGGTTTCGGGTGACCTTG |
| Mouse CCL5 | ATATGGCTCGGACACCACTC | TCCTTCGAGTGACAAACACG |
| Mouse CXCL1 | GCTGGGATTCACCTCAAGAA | TGGGGACACCTTTTAGCATC |
| Mouse PPARg | ttttcaagggtgccagtttc | aatccttggccctctgagat |
| Mouse TFF2 | Tgctttgatcttggatgctg | ggaaaagcagcagtttcgac |
| Human Cav1 | TCTCTACACCGTTCCCATCC | CAGGTCGATCTCCTTGGTGT |
| Human b2M | TGCTGTCTCCATGTTTGATGTATCT | TCTCTGCTCCCCACCTCTAAGT |
| Human IL-8 | GTGCAGTTTTGCCAAGGAGT | CTCTGCACCCAGTTTTCCTT |
| Human DLC1v1 | CAGGCACACCAACAAACCTGCG | GGCACAAGGCTCATCCTCGTCTG |
| Human DLC1v4 | ttgaagccaaggaagcttgt | tcgtctgaatcgtcacttcg |
| Human ACS | ATTGACTTGTGGTGGCATGA | CTGTGTGAACCACACCCTTG |
| Human HMGCoAS | CAAAAAGATCCATGCCCAGT | AAAGGCTTCCAGGCCACTAT |
| Human HMGCoAR | GGCATTTGACAGCACTAGCA | GCTGGAATGACAGCTTCACA |
| Human LDLR | GCGAAAGAAACGAGTTCCAG | TGACAGACAAGCACGTCTCC |
| Dog b2M | GCTACGTGTCAGGGTTCCAT | CATGCTTTACACGGCAGCTA |
| *H. pylori* CagA | agcaaaaagcgaccttgaaa | gggttccattcacaccattc |
| *H. pylori* UreB | CAACAAATCCCTACAGCTTTTGC | CCATCAGCAGGGCCAGTT |

DNA-sequences of 5’-(forward) and 3’-(reverse) primers for detection of human, mouse and *H. pylori* genes by RT-qPCR are listed. Oligonucleotide sequences from the human *CAV1* gene promoter containing SREs were used for performance of EMSA and ChIP assays.
