# Supplementary material for: Caveolin-1 Protects B6129 Mice against Helicobacter pylori Gastritis
Source: PLoS Pathog. 2013 Apr 11;9(4):e1003251. doi: 10.1371/journal.ppat.1003251 (PMC3623771; doi:10.1371/journal.ppat.1003251)
Supplement: Table S2 — DLC1 peptides identified by MALDI-MS. The amino acid sequence and the localization of peptides precipitated in CoIP experiments using Cav1 as a bait are presented. Peptides overlapped with both variant 1 and variant 4 of the human DLC1 protein. * Mascot total ion score by GPS Explorer 2 software; # Location of peptides identified by MALDI-MS in the coding sequence (CDS) of human DLC1 protein variants. (DOC) [file ppat.1003251.s004.doc]

**Table S2 DLC1 peptides identified by MALDI-MS.**

| **Acc. Nr.** | **Mass (Da)** | **Score *** | **Description** | **Species** |
| --- | --- | --- | --- | --- |
| Q96QB1 | 172.197 | 57 | **Rho GTPase-activating protein 7**  **Deleted in liver cancer 1 protein** | HUMAN |
| **Mass (Mr)** | **Location within CDS #** | | **Peptide sequence** | |
| 13.076.805  13.086.750  13.176.099  13.656.580  14.347.654  14.757.583  15.477.770  15.677.490  15.706.770  16.348.962  16.399.114  19.939.779  21.990.396  22.841.296  28.733.872  29.154.954 | DLC1 mRNA start of variant 4 (short form)  **DLC1 mRNA variant 1 (long form)**  DLC1 mRNA variant 1 and 4  DLC1 mRNA variant 1 and 4  DLC1 mRNA variant 1 and 4  DLC1 mRNA variant 1 and 4  DLC1 mRNA variant 1 and 4  DLC1 mRNA variant 1 and 4  DLC1 mRNA variant 1 and 4  **DLC1 mRNA variant 1 (long form)**  DLC1 mRNA variant 1 and 4  **DLC1 mRNA variant 1 (long form)**  DLC1 mRNA variant 1 and 4  DLC1 mRNA variant 1 and 4  DLC1 mRNA variant 1 and 4  **DLC1 mRNA variant 1 (long form)** | | R.LNTLNKCAVMK.L  **K.AEDGMQCLQLK.E**  K.SFGHLCAAEVVK.I  R.TGSFHGPGHISLR.R  K.HGFSWAVPKFMK.R  K.GMQRIVNQWSEK.F  K.DGPSPGGTLMDLSER.Q  K.LFQVPEEMSRCR.N  R.TGQPLPQSIQQAMR.Y  **K.SLELCNEISLSEIK.D**  R.WSRLEEFDVFSPK.Q  **K.DIAPEKQLLNSAVIAQQR.R**  K.DLNENLAATQGLAHMIAECK.K  K.FSDEGDSDSALDSVSPCPSSPK.Q  R.SVSNSTQTSSSSSQSETSSAVSTPSPVTR.T  **K.LDQLDQDIENALSTSSSPSGTPTNLRR.H** | |

The amino acid sequence and the localization of peptides precipitated in CoIP experiments using Cav1 as a bait are presented. Peptides overlapped with both variant 1 and variant 4 of the human DLC1 protein. * Mascot total ion score by GPS Explorer 2 software; # Location of peptides identified by MALDI-MS in the coding sequence (CDS) of human DLC1 protein variants.
